# Role of CD28^+^ PD-1^+^ Tc cells in immune response and prognosis prediction in hepatocellular carcinoma

**DOI:** 10.3389/fimmu.2025.1576193

**Published:** 2025-06-04

**Authors:** Wuhan Yang, Teng Pan, Yaowen Chen, Hao Guo, Yaqi Peng, Chao Wang, Li Peng, Shubin Wang

**Affiliations:** ^1^ Department of Hepatobiliary Surgery, The Fourth Hospital of Hebei Medical University, Shijiazhuang, China; ^2^ Department of Oncology, Shijiazhuang First Hospital, Shijiazhuang, China; ^3^ Department of General Surgery, The Second Hospital of Hebei Medical University, Shijiazhuang, China; ^4^ Department of General Medicine, The Fourth Hospital of Hebei Medical University, Shijiazhuang, China

**Keywords:** hepatocellular carcinoma, t cell exhaustion, CD28, PD-1, single-cell RNA-seq, tumor microenvironment

## Abstract

**Background:**

CD28^+^PD-1^+^ Tc cells (CD8^+^ T cells) constitute a dysfunctional subset of T cell; however, the mechanisms underlying their dysfunction and their significance in hepatocellular carcinoma (HCC) remain unclear. We aimed to elucidate the prognostic significance and molecular characteristics of CD28^+^PD-1^+^ Tc cell infiltration in HCC.

**Methods:**

We established a single-cell HCC transcriptional map, focusing on cell-cell communication and trajectory analysis of CD28^+^PD-1^+^ Tc cells. We assessed the correlation between CD28^+^PD-1^+^ Tc-cell enrichment and prognosis and investigated potential molecular mechanisms using enrichment analyses. Flow cytometry was used to compare CD28^+^PD-1^+^ Tc-cell infiltration between HCC and adjacent normal tissues and cytotoxic factors and immune checkpoint expression were evaluated.

**Results:**

Overall, 25,644 T cells were identified from single-cell RNA sequencing data from 10 HCC samples and corresponding normal samples. Overall T-cell infiltration was lower in HCC tissues, with significantly higher CD28^+^PD-1^+^ Tc-cell infiltration. Bulk RNA sequencing data integration revealed a correlation between higher CD28^+^PD-1^+^ Tc-cell infiltration and significantly worse prognosis. Flow cytometry confirmed higher CD28^+^PD-1^+^ Tc-cell enrichment in HCC tissues. Additionally, cytotoxic factor expression was significantly lower in CD28^+^PD-1^+^ Tc cells than in CD28^-^PD-1^+^ Tc cells, with lower expression of TIGIT and TIM-3 immune checkpoint molecules.

**Conclusions:**

Significantly high CD28^+^PD-1^+^ Tc-cell enrichment in HCC indicates potential immune dysfunction. CD28^+^PD-1^+^ Tc-cell enrichment may serve as a sensitive prognostic marker and indicator for predicting treatment responses.

## 1 Introduction

Liver cancer is the fourth leading cause of cancer-related deaths and the sixth most frequently diagnosed cancer annually (1). Hepatocellular carcinoma (HCC) accounts for 85–90% of primary liver cancers. At onset, HCC is often insidious, and it lacks typical early symptoms, with most patients having missed the optimal treatment window at the time of diagnosis (2). This delay contributes to its low 5-year survival rate and poor prognosis. Immunotherapy has emerged as a promising strategy for treating HCC; however, HCC’s complex tumor microenvironment (TME) and high heterogeneity significantly contribute to resistance and recurrence (3, 4). A comprehensive understanding of this TME heterogeneity is necessary for developing effective treatment strategies.

The TME comprises various immune and stromal cells that contribute to HCC progression (5). Cytotoxic CD8^+^ T cells are abundant within the TME and play a pivotal role in anti-tumor immunity by secreting granzyme B and interferon-γ (IFN-γ) to eradicate tumor cells (6). However, prolonged antigenic stimulation, coupled with the TME’s immunosuppressive characteristics, results in a gradual decline in immune efficacy. CD8^+^ T-cell functionality decline is characterized by reduced immune effector functions and heightened inhibitory receptor expression, particularly programmed cell death protein 1 (PD-1), culminating in T-cell exhaustion (7, 8). Exhausted CD8^+^ T cells rely on CD28 co-stimulatory signals for self-renewal and play a pivotal role in tumor immune evasion (9–11). Therefore, examining the characteristics and roles of CD8^+^ T-cell subsets within the TME is instrumental in predicting patient prognosis and establishing personalized therapeutic approaches.

CD28^+^PD-1^+^ Tc cells (CD8^+^ T cells) represent a state of functional exhaustion of CD8^+^ T cells caused by prolonged antigenic stimulation. These cells constitute a key component of immune evasion, particularly in tumor immunity and chronic viral infections. Continuous immune stimulation prompts certain CD8^+^ T cells to transition into CD28^+^PD-1^+^ Tc cells within HCC. These cells continue to express CD28, suggesting retained activation potential under certain conditions; however, their immune function is impaired by PD-1 expression, resulting in a loss of cytotoxicity and proliferation capacity (12–14) and rendering them less effective at eliminating tumor cells. CD28^+^ PD-1^+^ Tc cells contribute to the exhausted T-cell reservoir and serve as a significant marker of immune suppression (15). However, the specific functions and mechanisms related to the CD28^+^ PD-1^+^ Tc-cell subset in HCC remain unclear.

Therefore, we employed single-cell RNA sequencing (scRNA-seq) to delineate different T-cell types and their distinctions within HCC samples. We used CellChat and pseudotime analysis to examine the characteristics and dynamic evolution of the CD28^+^PD-1^+^ Tc-cell subset and integrated RNA-seq data from The Cancer Genome Atlas (TCGA) to predict CD28^+^PD-1^+^ Tc-cell infiltration in HCC and their association with patient prognosis. Moreover, we explored potential molecular mechanisms using enrichment and immune infiltration analyses. Finally, we confirmed the presence of these cells in HCC tissues and assessed their cytotoxic functions and immune checkpoint expression using flow cytometry.

## 2 Materials and methods

### 2.1 Transcriptomic data download and organization

The utilized datasets were primarily sourced from TCGA databases (https://portal.gdc.cancer.gov/). We employed the “TCGAbiolinks” package to download whole-genome expression profiles in TPM format and obtained clinical and single nucleotide variation data predicted using the “VarScan2 Variant Aggregation and Masking” tool for HCC (16). Overall, 374 tumor and 50 normal samples (n = 424) from the TCGA-LIHC project were included.

### 2.2 Single-cell sequencing data processing

We obtained scRNA-seq datasets from HCC from the Gene Expression Omnibus database, comprising tissue samples from 10 HCC cases. The filtered single-cell data from GSE140228 were imported using the Seurat package (17). Clustering was performed using the “FindClusters” tool, resulting in 18 clusters across 21 principal component components, with a resolution of 0.2. The “RunUMAP” function was applied to validate cell aggregation. Differentially expressed genes (DEGs) in each cell cluster were identified using the “FindAllMarkers” function. Subsequently, cell types were classified based on specific biomarkers, and the proportions of each type were calculated. The sub-clustering process for T cells followed the same methodology as that for the entire cell dataset.

### 2.3 Constructing cell trajectories using pseudotime analysis

We conducted pseudotime analysis using Monocle 2 to construct cellular trajectories and identify key gene expression programs driving HCC progression (18). Regarding T-cell pseudotime analysis, the raw count data underwent normalization via size factors estimated for trajectory inference. Trajectories were constructed using genes exhibiting both high expression and significant variability (dispersions ≥ 1 and average expressions ≥ 0.1) (19). The default DDRTree algorithm parameters were employed for this purpose. Branching Expression Analysis Modeling feature in Monocle 2 was utilized to pinpoint genes significantly dependent on branching expression (18). This process allowed us to elucidate the transcriptional features of HCC cells in different states. Additionally, we explored the genes determining different transcriptional states, with a focus on analyzing gene expression differences in T-cell trajectories between HCC and normal tissues.

### 2.4 Cell-cell communication analysis and receptor expression

The “CellChat” package was used to create CellChat objects from UMI count matrices of HCC and adjacent tissue samples (https://www.github.com/sqjin/CellChat) (20). We performed intercellular communication analysis using the “CellChatDB.human” database. The “mergeCellChat” tool merged the CellChat objects from each group to facilitate comparisons of the total number and intensity of interactions. Differences in interaction quantities or intensities among various cell types across groups were visualized using the “netVisual_diffInteraction” tool. The “netVisual_bubble” and “netVisual_aggregate” tools were utilized to depict the distribution of signaling gene expressions across groups.

### 2.5 Enrichment analyses

We used the “clusterProfiler” package to conduct Gene Ontology (GO) analysis (encompassing biological processes, molecular functions, and cellular components) and Kyoto Encyclopedia of Genes and Genomes enrichment analyses on DEGs (*P* < 0.05) (21–23). The intersection of DEGs identified in CD28^+^ PD1^+^ Tc cells and HCC samples was considered the set of DEGs associated with CD28^+^ PD1^+^ Tc cells in HCC.

### 2.6 Survival analysis

The CD28^+^PD-1^+^ T-cell enrichment score was calculated based on marker genes identified in GSE140228 and was inferred in TCGA-LIHC samples using the single-sample gene set enrichment analysis (ssGSEA) method (24). We quantified the relative enrichment scores of these cell types based on each sample’s gene expression profiles to assess the prognostic value of significantly upregulated genes associated with CD28^+^PD-1^+^ Tc cells in HCC. Enrichment scores for upregulated gene sets associated with specific cell types were calculated using ssGSEA. The “surv_cutpoint” tool was employed to select the optimal cutoff value, classifying samples into low and high-enrichment groups for the specific cell type. Kaplan–Meier survival curves were constructed, and log-rank tests were performed to assess statistical significance. Time-dependent receiver operating characteristic curves were utilized to assess the predictive efficacy of the enrichment score.

### 2.7 Nomogram construction and validation

We extracted clinical data, including sex, age, and tumor staging, from patients in the TCGA cohort. Age was categorized using 65 years as the cutoff (25). Cox regression analyses were conducted by integrating clinical data (such as age, sex, T stage, N stage, M stage) with enrichment scores derived from the survival analyses. Multivariate Cox regression analysis identified factors with statistical significance (*P* < 0.05) for inclusion in the prognostic model. A nomogram was developed using the “RMS” package to predict overall survival at 1, 3, and 5 years. Calibration curves were employed to evaluate the nomogram’s accuracy.

### 2.8 Gene set variation analysis and gene set enrichment analysis

Differential expression analysis was performed between high and low-enrichment groups using the “limma” package (26). Gene set enrichment analysis (GSEA) was subsequently performed using the “clusterProfiler” package. The “GSVA” package was utilized to conduct gene set variation analysis (GSVA) to examine the biological functional discrepancies between low- and high-enrichment cohorts. The results were visualized using the “pheatmap” package. We referenced the “c2.cp.kegg.v7.5.1.symbols” gene set derived from the Molecular Signatures Database (27–29).

### 2.9 Immune infiltration analysis

Data on 28 distinct immune cell types were sourced from the Tumor and Immune System Interactions Database for the immune infiltration analysis (http://cis.hku.hk/TISIDB/index.php). This method integrates multiple Gene Expression Omnibus datasets, including GSE140228, for deconvolution-based inference (30). Differences in immune cell infiltration between high- and low-enrichment groups for specific cell types were illustrated using the “ggplot2” package (31).

### 2.10 Drug sensitivity analysis

Half-maximal inhibitory concentration (IC50) data and corresponding gene expression profiles were obtained from the Genomics of Drug Sensitivity in Cancer database (https://www.cancerrxgene.org/) (32). The “oncoPredict” package was employed to forecast potential drug sensitivity in patients with varying levels of specific cell type enrichment in HCC (33).

### 2.11 Tumor mutation burden

Mutation data were analyzed to illustrate the landscape of genomic variations. Somatic variations, including single nucleotide polymorphisms, insertions and deletions, the tumor mutation burden (TMB), and mutation frequency, were presented using the “maftools” package (34). The top 20 frequently mutated genes are generally considered principal driver genes in malignancies (35).

### 2.12 Tumor immune dysfunction and exclusion

We conducted a Tumor Immune Dysfunction and Exclusion (TIDE) analysis (http://tide.dfci.harvard.edu/) to assess the immunotherapeutic response (36). Immune checkpoints constitute molecules on immune cells that modulate levels of immune activation. We compared immune checkpoint gene expression between the two groups.

### 2.13 Clinical sample collection

We collected nine pairs of HCC samples and corresponding adjacent tissues from the Fourth Hospital of Hebei Medical University (Shijiazhuang, China). The inclusion criteria included patients diagnosed with primary HCC undergoing their first treatment who had received partial hepatectomy and had not undergone interventional therapy, targeted therapy, or immunotherapy before surgery. The exclusion criteria included individuals with a history of other malignancies. This research was approved by the hospital’s ethics committee (No. 2023KS181) and was conducted following the principles outlined in the Declaration of Helsinki, with informed consent acquired from all participants.

### 2.14 Magnetic bead separation and CD8^+^ T-cell enrichment

A tissue digestion solution was prepared, and a single-cell suspension was obtained by filtering through a mesh. The suspension was centrifuged at 500×g for 10 min, and the pellet was retained and washed 1–2 times with pre-cooled phosphate-buffered saline. Lymphocytes were isolated using the Percoll method. In total, 1×10⁷ lymphocytes were resuspended in 150 µL of buffer, gently mixed with 50 µL of CD3 magnetic beads, incubated, washed, and resuspended. Next, 3 mL of the cell suspension was added to an LS column to collect CD3^+^ lymphocytes (T-cells). The same steps were repeated to enrich CD8^+^ T cells, which were counted and reserved for further use. The sorted CD8⁺ T cells were considered to be αβ T cells rather than γδ T cells. This procedure aimed to detect the ratios of CD28^−^PD-1^+^ and CD28^+^PD-1^+^ Tc-cell subsets in HCC tissues relative to normal tissues.

### 2.15 Flow cytometry

CD8^+^ T cells enriched using magnetic beads were stained with surface antibodies for CD8, PD-1, and CD28, followed by flow cytometry. Voltage adjustments were made using a blank tube, and compensation was set using single-stained tubes before running the sample tubes. CD28^+^PD-1^+^ and CD28^-^PD-1^+^ Tc cells were sorted and analyzed using flow cytometry to determine the proportion of CD28^+^PD1^+^ Tc cells in HCC and normal tissues. After sorting CD28^+^PD-1^+^ and CD28^−^PD-1^+^ Tc cells from HCC tissues, the cells were resuspended in buffer, and 1 µL of eBioscience cell stimulation components was added. The cells were incubated at 37°C for 3 h to facilitate their activation and subsequent cytokine secretion. After incubation, anti-PD-1 and anti-CD28antibodies were used for staining. The cells were incubated in the dark at room temperature for 30 min and subsequently washed to remove unbound antibodies. Next, cells were treated with eBioscience intracellular fixation and permeabilization buffer and stained with antibodies against tumor necrosis factor α (TNF-α), IFN-γ, granzyme B, perforin, CTLA4, TIM3, and TIGIT. Flow cytometry was performed, with voltage and compensation adjusted as previously described.

Specific antibody details and information regarding the microbeads are provided in **Supplementary Table S14**, and gating strategies are provided in “**Supplementary Material**”. An Attune NXT flow cytometer (Thermo Fisher Scientific, Waltham, MA, USA) was used for detection, and data were analyzed using FlowJo V10 software (Treestar, San Carlos, CA, USA).

### 2.16 Real-time PCR

Total RNA was extracted from the samples using the Trizol reagent, and RNA quality and concentration were assessed using a BioPhotometer (Eppendorf, Eppendorf, Germany) to ensure RNA integrity. Reverse transcription was performed using the HiFiScript gDNARemoval cDNA Synthesis Kit (CW2582M, CWBio, Taizhou, China), converting RNA into complementary DNA. The target gene and reference gene primers (**Supplementary Table S14**) were used for amplification. Reverse transcription-PCR (RT-PCR) was conducted using ChemoHS qPCR Mix (No ROX, MQ00101S, Monad, Suzhou, China) according to the manufacturer’s protocol. Relative mRNA expression levels were calculated using the 2^−ΔΔCT^ method for data analysis.

### 2.17 Enzyme-linked immunosorbent assays

Supernatant samples from CD28^+^PD-1^+^ T cells and CD28^−^PD-1^+^ T cells were collected and analyzed using the Human Enzyme-Linked Immunosorbent Assay (ELISA) Kit (**Supplementary Table S14**) to measure the levels of Granzyme B, IFN-γ, Perforin, and TNF-α, following the instructions provided in the kit. Statistical analysis was performed using t-test, with *P* < 0.05 considered statistically significant. All data were processed using Microsoft Excel 2016 (Microsoft Corp., Redmon, WA, USA).

### 2.18 Statistical analysis

R (version 4.1.2) was utilized for statistical analyses. Comparisons of categorical data between the two groups were performed using the chi-squared test. Kaplan–Meier curves and log-rank tests were used to compare survival rates, utilizing the “survminer” package to generate survival curves. Univariate and multivariate Cox regression analyses were performed to assess prognostic variables. Data visualization was performed using the “ggplot2” package, and heatmaps were generated using the “Pheatmap” package. We utilized t-tests or one-way analysis of variance to determine significant differences in normally distributed data. Wilcoxon or Kruskal–Wallis tests were used for non-normally distributed data. The threshold for statistical significance was set at *P* < 0.05.

## 3 Results

### 3.1 Single-cell dimensionality reduction, clustering, and annotation

**Figure 1:**
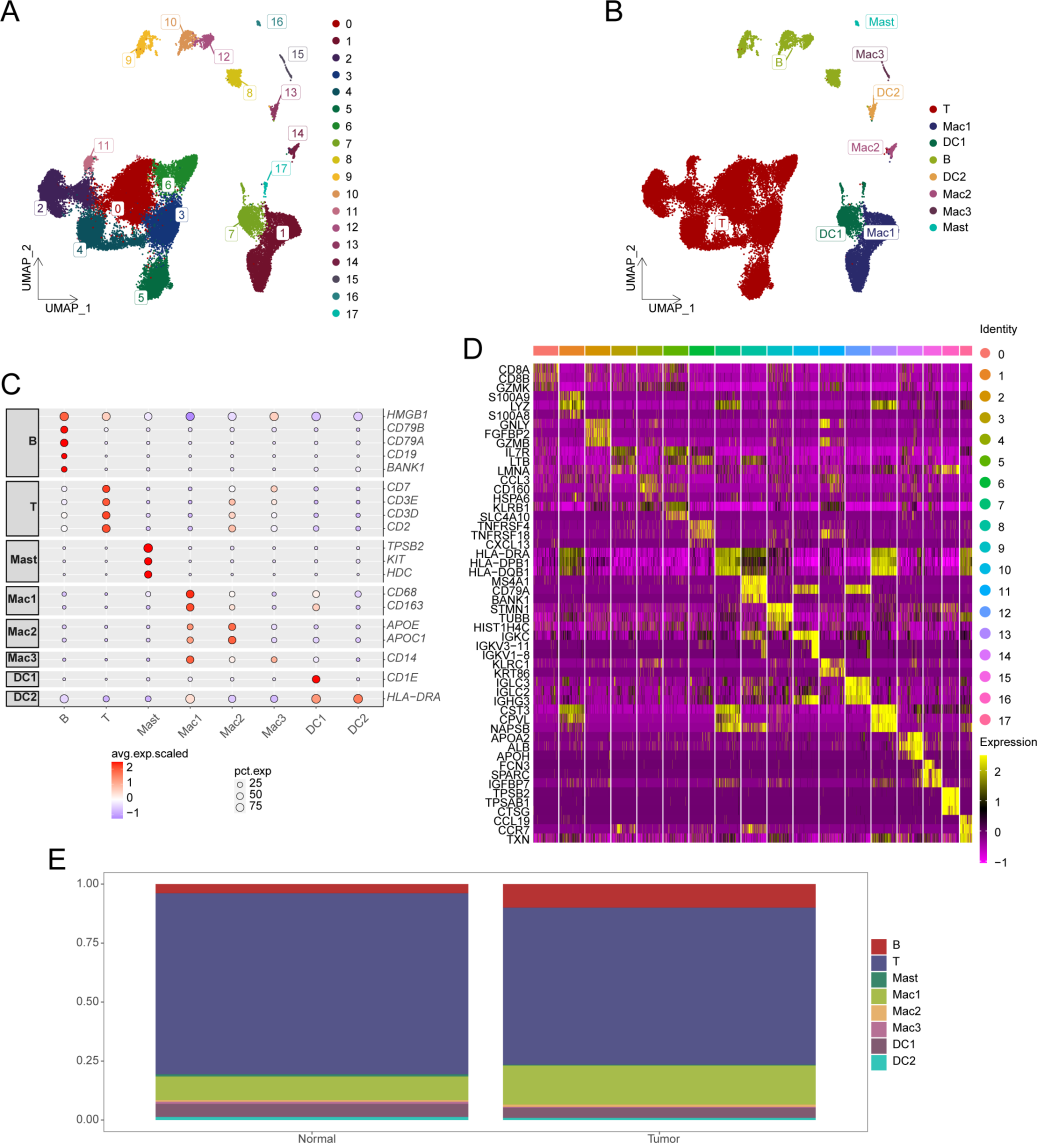
Identification of cell subsets from single-cell sequencing data. **(A)** UMAP plot displaying the distribution of HCC cell subsets. **(B)** UMAP plot showing annotation results for HCC cell subsets. **(C)** Expression of marker genes in each cell type. **(D)** Heatmap illustrating cell group-specific expressed genes. **(E)** Cumulative histogram showing the distribution of cell types in HCC and normal tissues. HCC, hepatocellular carcinoma; UMAP, Uniform Manifold Approximation and Projection.

Overall, 36,193 cells were extracted from the single-cell transcriptome and subsequently clustered into 18 distinct clusters (**Figure 1A**), with cell types annotated according to gene expression profiles and cell-specific biomarkers (**Supplementary Table S1**). **Figure 1B** illustrates the identification of eight unique cell types. The expression patterns of specific genes corresponding to each cell type are represented using dot plots (**Figure 1C**), whereas the expressions of three characteristic genes for each cell group are illustrated using a heatmap (**Figure 1D**). **Figure 1E** depicts the proportions of different T-cell types in HCC samples compared to normal controls. We observed a significant reduction in T-cell abundance in HCC samples (*P* < 0.05).

### 3.2 Gene expression characteristics of T-cells

**Figure 2:**
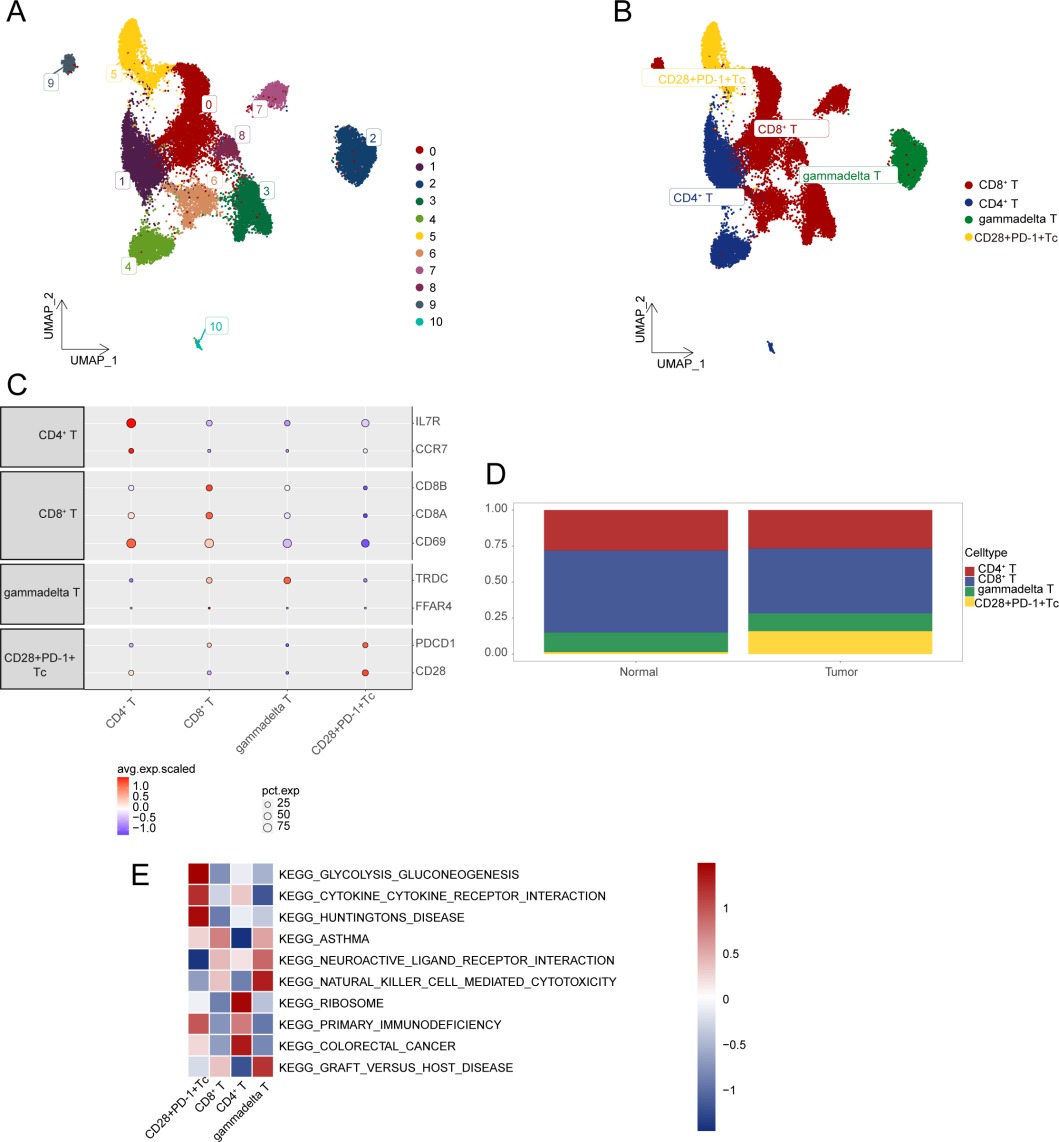
Characterization of gene expression profiles within T-cell subsets. **(A)** UMAP plot illustrating the distribution of T-cell subsets. **(B)** UMAP plot displaying annotation results for T-cell subsets. **(C)** Expression levels of marker genes across various cell types. **(D)** Cumulative histogram showing the distribution of T-cell subpopulations in HCC and normal tissues. **(E)** Visualization of GSVA analysis results through heatmap. HCC, hepatocellular carcinoma; UMAP, Uniform Manifold Approximation and Projection; GSVA, gene set variation analysis.

Overall, 25,644 T cells were obtained and re-clustered into 11 clusters (**Figure 2A**), with cell types annotated based on gene expression profiles and cell-specific biomarkers (**Supplementary Table S2**). Four distinct T-cell subsets were identified, including CD28^+^PD-1^+^ Tc cells, γδ T cells, CD4^+^ T cells, and CD8^+^ T cells. The CD28⁺PD-1⁺ Tc cells referenced here represent αβ CD8⁺ T cells, rather than γδ T cells (**Figure 2B**). Dot plots were used to visualize specific genes associated with each T-cell subset (**Figure 2C**). The comparative proportions of various T-cell subsets in HCC samples against normal tissues are illustrated in **Figure 2D**, the proportion of CD28^+^PD-1^+^ T cells was significantly higher in HCC tissues than in normal tissues (*P* < 0.05). GSVA was performed to select the three most significantly different pathways for each T-cell subset to create a pathway activity heatmap (**Figure 2E**; **Supplementary Table S3**).

### 3.3 Pseudotime analysis

Analysis of transcriptional states revealed five distinct T-cell states, each corresponding to a specific transcriptional phase (**Supplementary Figures S1A–C**). **Supplementary Figure S1D** illustrates the proportions of different T-cell states in HCC and normal samples, revealing a higher proportion of certain T-cell states within the HCC samples. We identified genes that define Monocle 2 in HCC cells to elucidate the molecular determinants responsible for these transitions. Genes highly expressed in the pre-branch phase were primarily enriched in GO biological processes, such as “T-cell activation”, “leukocyte cell-cell adhesion”, and “regulation of T-cell activation”. Genes associated with “positive regulation of lymphocyte activation” were highly expressed in Branch 1, whereas those enriched in “cytoplasmic translation”, “ribosome biogenesis”, and “ribonucleoprotein complex biogenesis” were dominant in Branch 2 (**Supplementary Figure S1E**). These findings suggest that the various T-cell states are closely associated with their function and progression in HCC. Pseudotime analysis demonstrated the dynamic transcriptional patterns of T cells in HCC and their significant differences from normal tissues.

### 3.4 Cell-cell communication analysis

The total number and strength of cellular interactions was higher in HCC than in normal controls (**Supplementary Figure S2A**), with most interactions exhibiting higher quantity and intensity (**Supplementary Figures S2B, C**). Signaling patterns were compared between normal and HCC tissues were compared, with the overall signaling patterns shown in **Supplementary Figure S3D**. For example, the *CXCL* signal intensity derived from CD28^+^PD-1^+^ Tc cells was different in HCC. Moreover, the *MHC-II* signal intensity acting on CD28^+^PD-1^+^ Tc cells and the *ITGB2* signal intensity from these cells were higher in HCC (**Supplementary Figures S3**, **S4**). We further analyzed potential interactions between CD28^+^PD-1^+^ Tc cells and other cell types. Significant signaling pathways identified in HCC included *HLA-E* and *CLEC2C*. Notably, the interaction between *HLA-E* expressed by CD28^+^PD-1^+^ Tc cells and the corresponding *NKG2C* receptor on γδ T cells was enhanced in HCC(**Supplementary Figure S5A**), whereas that between *CLEC2C* from CD28^+^PD-1^+^ Tc cells and *KLRB1* on CD4^+^ T cells was lower in HCC (**Supplementary Figure S5B**). The interaction between the *LGALS9* ligand from DC2 cells and its CD28^+^PD-1^+^ Tc cell receptor was higher in HCC (**Supplementary Figure S6A**), whereas that between the *MIF* from Mac3 and its CD28^+^PD-1^+^ Tc cell receptor was lower in HCC (**Supplementary Figure S6B**). Furthermore, *CD69* expression in CD28^+^PD-1^+^ Tc cells was significantly lower in HCC, whereas the receptor *KLRB1* showed low expression in CD4^+^ T cells. This may explain the development of the *CLEC2C* pathway involving both CD28^+^PD-1^+^ Tc cells and CD4^+^ T cells in HCC (**Supplementary Figure S7A**). The receptor *NKG2C* for *HLA-E* was highly expressed in γδ T cells in HCC, elucidating the formation of the *HLA-E* pathway involving both CD28^+^PD-1^+^ Tc cells and γδ T cells in HCC (**Supplementary Figure S7B**).

### 3.5 Enrichment analysis of DEGs related to CD28^+^PD-1^+^ Tc cells

We identified 1,222 DEGs (*P* < 0.05 and |Log2FC| > 0.25) between CD28^+^PD-1^+^ Tc cells and other cell subtypes in HCC (**Supplementary Table S4**). The heatmap displays the top 10 upregulated genes in CD28^+^PD-1^+^ Tc cells (**Supplementary Figure S8A**). A comparison between HCC and normal tissues revealed 3,293 DEGs with significant differences between the two groups (*P* < 0.05, |Log2 fold change| > 1, **Supplementary Table S5**). The heatmap presents the top five upregulated (*PLVAP*, *DIPK2B*, *CD34*, *TOMM40L*, *SLC26A6*) and downregulated (*CLEC4M*, *CLEC1B*, *STAB2*, *BMP10*, *BMPER*) genes in HCC samples (**Supplementary Figure S8B**). We identified 222 key genes that intersected among the DEGs from both groups (**Supplementary Figure S8C**, **Supplementary Table S6**). The GO results (**Supplementary Table S7**) revealed that these genes were enriched in biological processes such as “cytoplasmic translation” (GO:0002181), “defense response to bacterium” (GO:0042742), and “activation of immune response” (GO:0002253), and cellular components such as “cytosolic ribosome” (GO:0022626), “ribosomal subunit” (GO:0044391), and “ribosome” (GO:0005840). Regarding molecular functions, the genes were enriched for “structural constituent of ribosome” (GO:0003735), “antigen binding” (GO:0003823), and “rRNA binding” (GO:0019843) (**Supplementary Figure S8D**). Kyoto Encyclopedia of Genes and Genomes analysis (**Supplementary Table S8**) revealed significant pathways, including “Ribosome” (hsa03010), “Coronavirus disease - COVID-19” (hsa05171), and “Salmonella infection” (hsa05132) (**Supplementary Figure S8E**).

### 3.6 Survival analysis and nomogram construction

**Figure 3:**
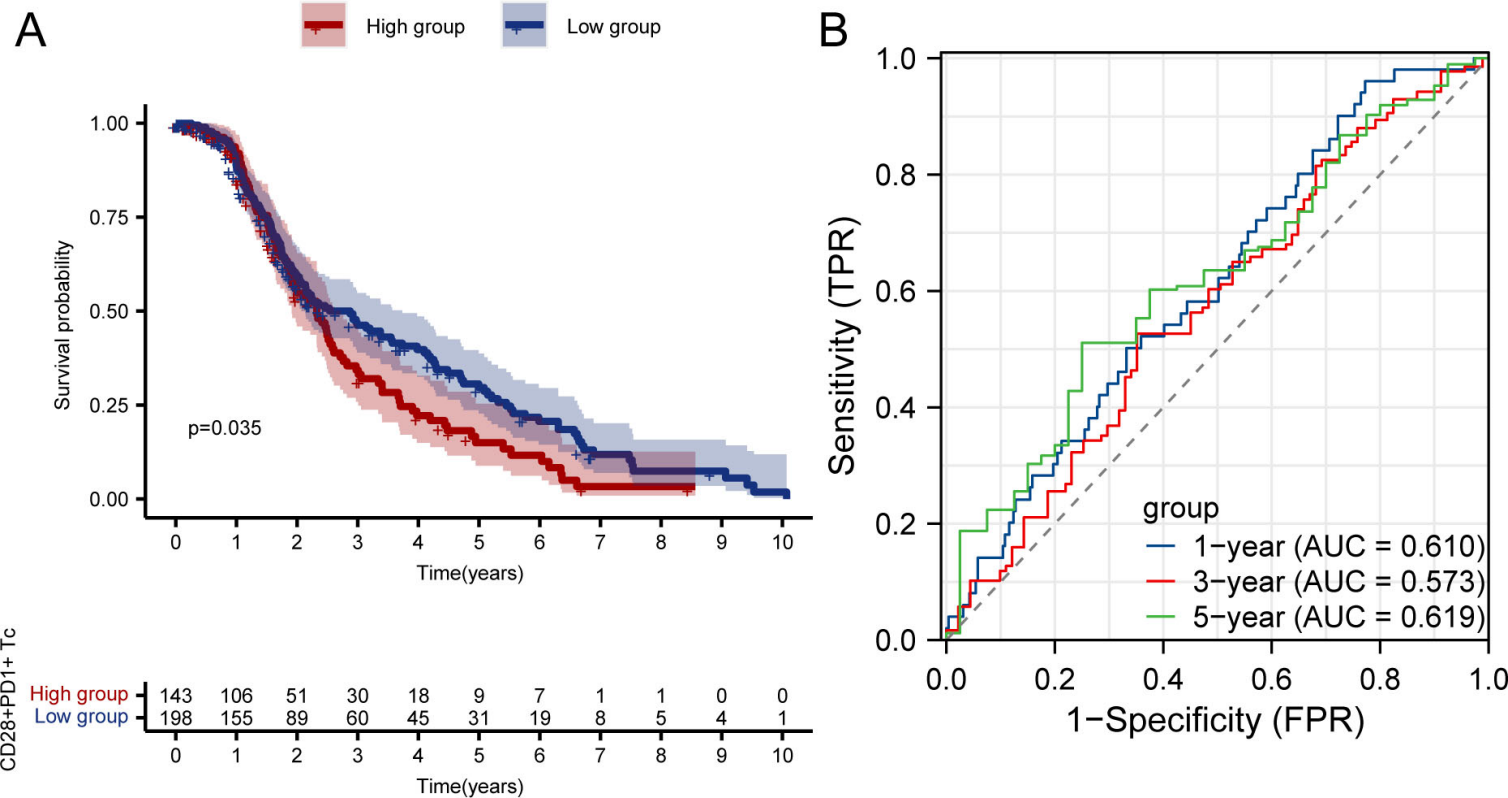
Survival analysis and ROC curve of high and low enrichment groups of CD28^+^PD-1^+^ Tc cells in patients with HCC. **(A)** Survival analysis of high and low enrichment groups of CD28^+^PD-1^+^ Tc cells in patients with HCC. **(B)** ROC curve validating the CD28^+^PD-1^+^ Tc-cell enrichment level. HCC, hepatocellular carcinoma; ROC, Receiver Operating Characteristic.

**Figure 4:**
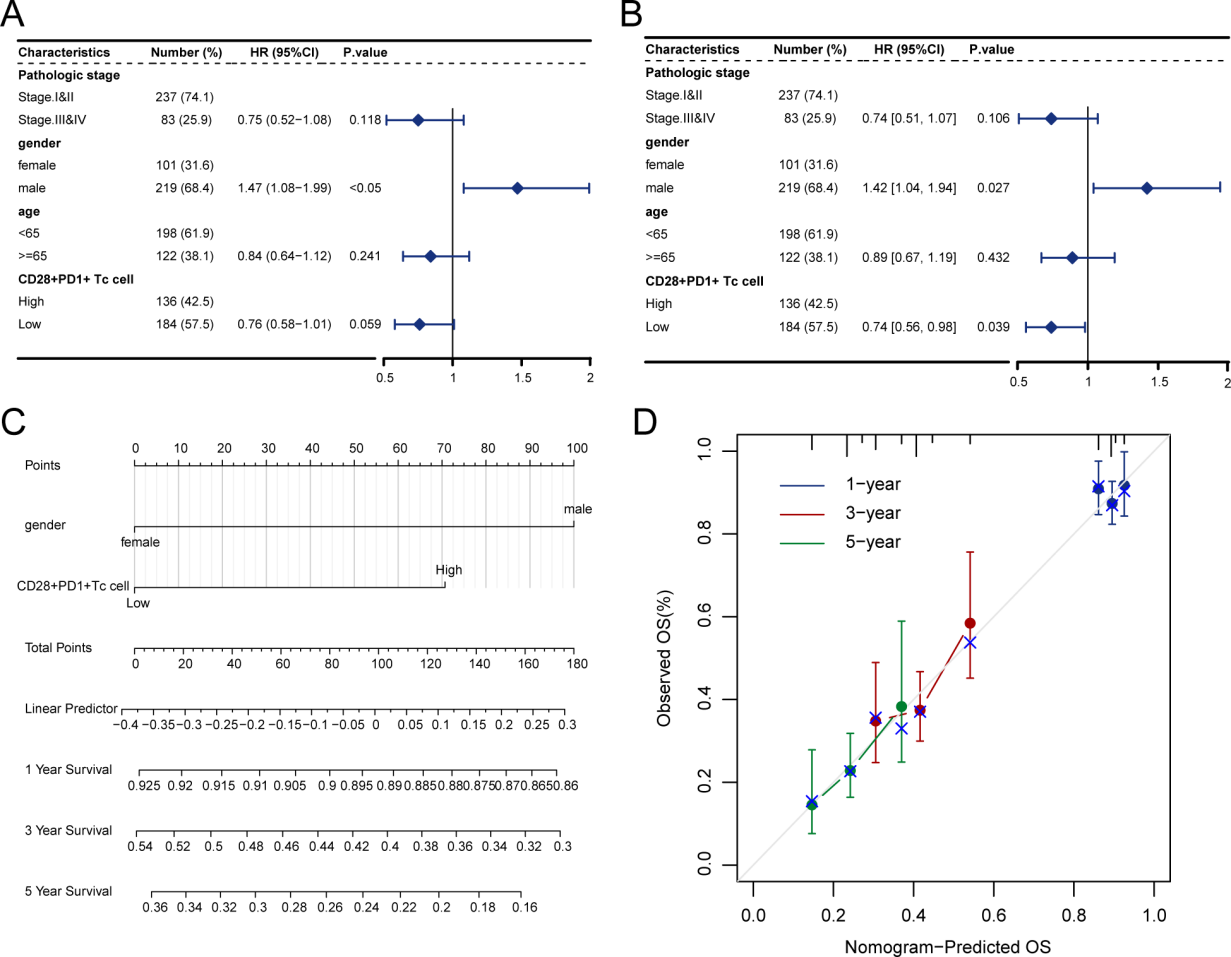
Enrichment score of CD28^+^PD-1^+^ Tc cells as an independent prognostic factor. **(A)** Forest plot illustrating the outcomes of univariate Cox regression analysis on clinical characteristics. **(B)** Forest plot depicting the results of multivariate Cox regression analysis on clinical characteristics. **(C)** Nomogram for predicting 1-, 3-, and 5-year survival rates. **(D)** Calibration curves for the nomogram at 1, 3, and 5 years.

Kaplan–Meier survival curves showed that patients with high CD28^+^PD1^+^ Tc-cell levels exhibited notably poorer prognosis than those with low levels (**Figure 3A**); the predictive performance of CD28^+^PD-1^+^ T-cell levels was evaluated using receiver operating characteristic curves (**Figure 3B**).Cox regression analysis indicated that the presence of CD28^+^PD-1^+^ Tc cells was an independent prognostic risk factor for patients with HCC (**Figures 4A, B**). We used multivariate Cox regression analysis to develop a nomogram, which demonstrated that risk scores could effectively forecast clinical outcomes (**Figure 4C**). Calibration curves showed that the nomogram was stable and accurate at 1, 3, and 5 years (**Figure 4D**).

### 3.7 GSEA and GSVA between high- and low-enrichment groups

We used the Molecular Signatures Database to identify the most important pathways according to normalized enrichment scores (NES) (**Supplementary Table S9**). The GSEA results showed that Ribosome (NES = 1.8991, adjusted P = 0.0157, FDR = 0.0085, **Supplementary Figure S9A**), Spliceosome (NES = 1.8342, adjusted P = 0.0157, FDR = 0.0085, **Supplementary Figure S9B**), and Cell Cycle (NES = 1.7758, adjusted P = 0.0157, FDR = 0.0085, **Supplementary Figure S9C**) were significantly enriched in the high-enrichment group. In contrast, Fatty Acid Metabolism (NES = −2.4043, adjusted P = 0.0297, FDR = 0.0162, **Supplementary Figure S9D**), Retinol Metabolism (NES = −2.4089, adjusted P = 0.0297, FDR = 0.0162, **Supplementary Figure S9E**), and Complement and Coagulation Cascades (NES = −2.4189, adjusted P = 0.0297, FDR = 0.0162, **Supplementary Figure S9F**) were significantly enriched in the low-enrichment group. Additionally, we used GSVA to create a heatmap of pathway activity for the five most significant pathways between the high and low-enrichment groups (**Supplementary Figure S9G**; **Table S10**).

### 3.8 Immune infiltration analysis

We used the ssGSEA method to analyze the infiltration levels of 28 immune cell types in high- and low-enrichment groups (**Supplementary Table S11**). The relative proportions of the 28 immune cell subpopulations were displayed in a histogram, revealing individual variability in immune cell ratios in HCC (**Supplementary Figure S10A**). Most immune cells were positively correlated with one another, notably with the enrichment score of CD28^+^PD-1^+^ Tc cells (**Supplementary Figure S10B**). The infiltration level of activated B cells was lower in the high-enrichment group, whereas those of activated CD4 T-cells, activated dendritic cells, effector memory CD4 T-cells, eosinophils, macrophages, memory B-cells, natural killer T-cells, plasmacytoid dendritic cells, T follicular helper cells, Type 17 T-helper cells, and Type 2 T-helper cells were significantly higher in the high-enrichment group (P < 0.05, **Supplementary Figure S11A**).

Furthermore, significant correlations were detected between DEGs in both groups and their corresponding immune cells (**Supplementary Figures S11B–J**). The genes *CDK1* (R = 0.733, *P* < 0.001, **Supplementary Figure S11B**), *RRM2* (R = 0.7486, *P* < 0.001, **Supplementary Figure S11C**), *CDKN3* (R = 0.7313, *P* < 0.001, **Supplementary Figure S11D**), *CCNB1* (R = 0.7318, *P* < 0.001, **Supplementary Figure S11E**), *PTTG1* (R = 0.7479, *P <*0.001, **Supplementary Figure S11F**), *KIF4A* (R = 0.7312, *P* < 0.001, **Supplementary Figure S11G**), *SPC25* (R = 0.7443, *P* < 0.001, **Supplementary Figure S11H**), *NEK2* (R = 0.7425, *P* < 0.001, **Supplementary Figure S11I**), and *KIFC1* (R = 0.7326, *P* < 0.001, **Supplementary Figure S11J**) were significantly positively correlated with activated CD4^+^ T cells.

### 3.9 TMB, drug sensitivity analysis, and TIDE

**Figure 5:**
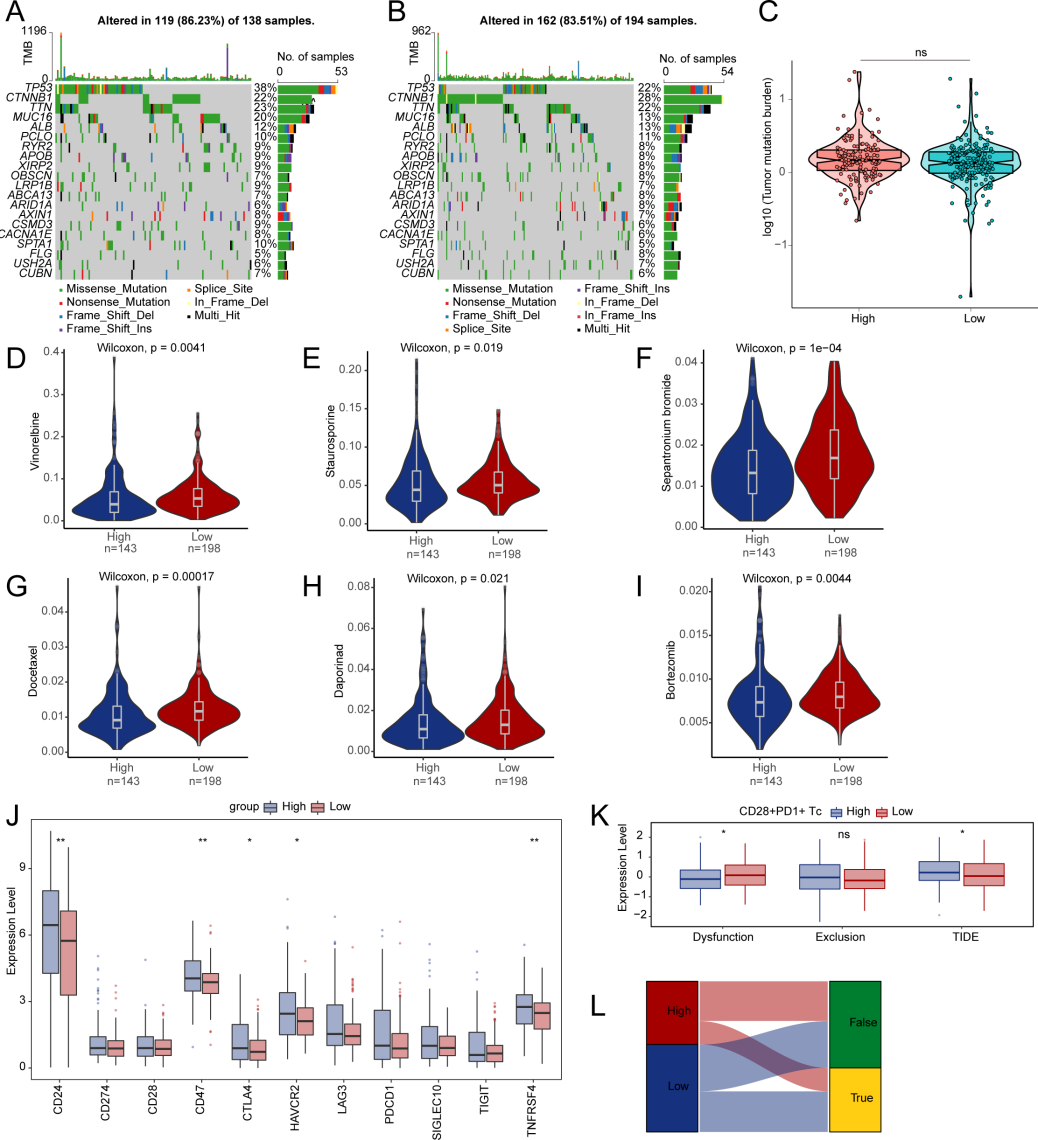
Differences in TMB, drug sensitivity, and TIDE between high- and low-enrichment groups. **(A, B)** The top 20 genes with the highest mutation frequencies in both groups. **(C)** Comparison of TMB between two enrichment groups. Differences in drug sensitivity for vinorelbine **(D)**, staurosporine **(E)**, sepantronium bromide **(F)**, docetaxel **(G)**, daporinad **(H)**, bortezomib **(I)** between two groups. **(J)** Boxplot depicting the expression of immune checkpoints between two groups. **(K)** Differences in immune therapy responses assessed by the TIDE prediction between two groups. **(L)** Sankey diagram evaluating immune therapy responses between high- and low-enrichment groups using the TIDE prediction. TMB, tumor mutation burden; TIDE, tumor immune dysfunction and exclusion. ns, p ≥ 0.05; *, p < 0.05; **, p < 0.01.

These analyses were performed based on grouping by CD28^+^PD-1^+^ Tc-cell enrichment levels, aiming to reveal their relationship with the TME, immune evasion, and therapeutic response. *TP53* showed the most frequent mutations in the high- and low-enrichment categories, followed by *CTNNB1* (**Figures 5A, B**). Analysis of somatic mutations related to HCC showed no significant difference in TMB between the high- and low-enrichment groups (P > 0.05) (**Figure 5C**). We examined whether the enrichment score of CD28^+^PD-1^+^ Tc cells could accurately predict chemotherapy sensitivity in patients with HCC, investigating the clinical efficacy of vinorelbine (**Figure 5D**), staurosporine (**Figure 5E**), sepantronium bromide (**Figure 5F**), docetaxel (**Figure 5G**), daporinad (**Figure 5H**), and bortezomib (**Figure 5I**) in HCC treatment (**Supplementary Table S12**). Individuals with high enrichment showed a heightened sensitivity to these chemotherapeutic agents, indicating chemotherapy as a promising treatment option.

Analysis of immune checkpoint expression between the two groups revealed an upregulation of the genes *CD24*, *CD47*, *CTLA4*, *HAVCR2*, and *TNFRSF4* in the high-enrichment group (**Figure 5J**). We used TIDE to evaluate the potential clinical efficacy of immune therapy across different enrichment subgroups (**Supplementary Table S13**). A higher TIDE score indicates a greater potential for immune evasion, which may be associated with reduced responsiveness to immune checkpoint blockade, as predicted by transcriptomic models. The subgroup with high enrichment had a higher TIDE score and exhibited significantly less functional impairment compared to the low-enrichment subgroup (**Figure 5K**). We observed a notable variation in immune therapy responses between the two groups, suggesting that the low-risk group is more likely to benefit from ICI treatment (**Figure 5L**).

### 3.10 Increased infiltration of CD28^+^PD-1^+^ Tc cells in HCC tissue

**Figure 6:**
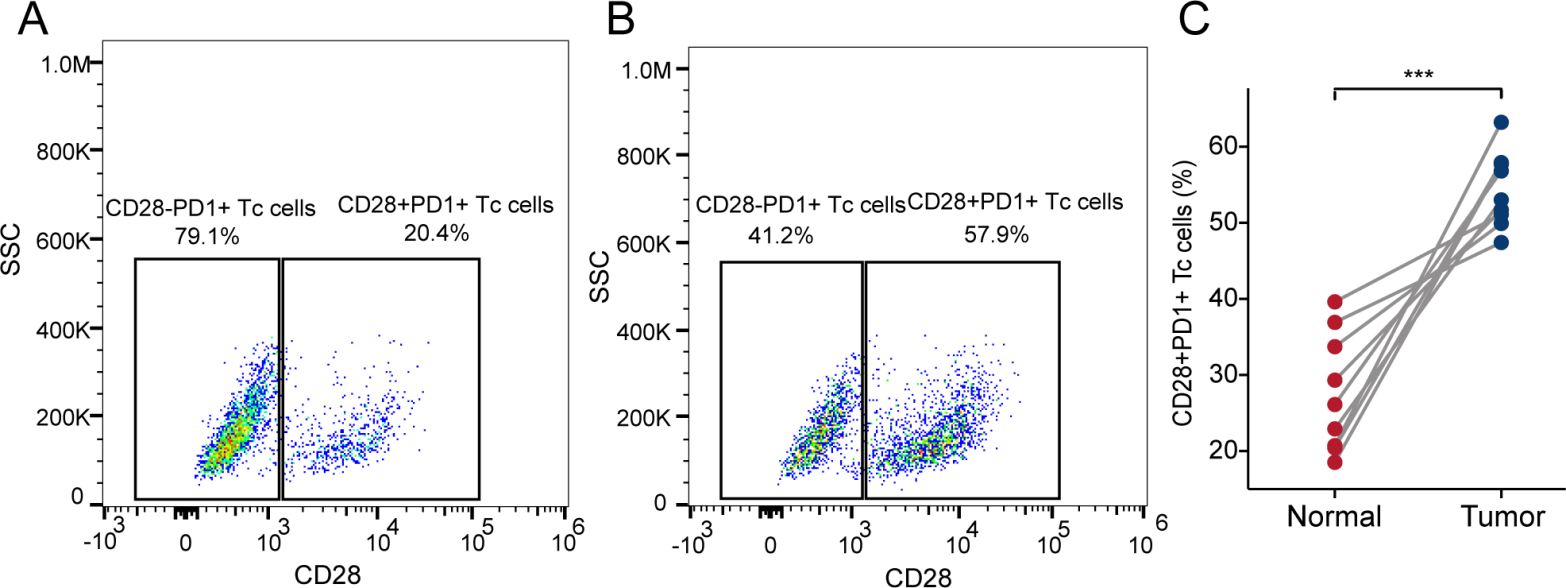
Infiltration of CD28^+^PD-1^+^ Tc cells in HCC and adjacent tissues. **(A)** Representative scatter plot showing the percentage of CD28^+^PD-1^+^ Tc cells *vs* CD28^-^PD-1^+^ Tc cells in adjacent tissues. **(B)** Representative scatter plot showing the percentage of CD28^+^PD-1^+^ Tc cells *vs* CD28^-^PD-1^+^ Tc cells in HCC tissues. **(C)** The percentage of CD28^+^PD-1^+^ Tc cells in HCC tissues was significantly higher than that in adjacent tissues (p< 0.001). HCC, hepatocellular carcinoma. ***, p < 0.001.

We collected nine paired samples of HCC and normal tissues for flow cytometric analysis. The results showed that CD28^+^PD-1^+^ Tc-cell infiltration was significantly higher in HCC tissues than in adjacent tissues (**Figure 6,** P < 0.001), which is consistent with the bioinformatics analysis, indicating that CD28^+^PD-1^+^ Tc cells were significantly enriched in HCC and may contribute to immune modulation in the HCC TME.

### 3.11 Decreased cytotoxic function of CD28^+^PD-1^+^ Tc cells in HCC tissue

**Figure 7:**
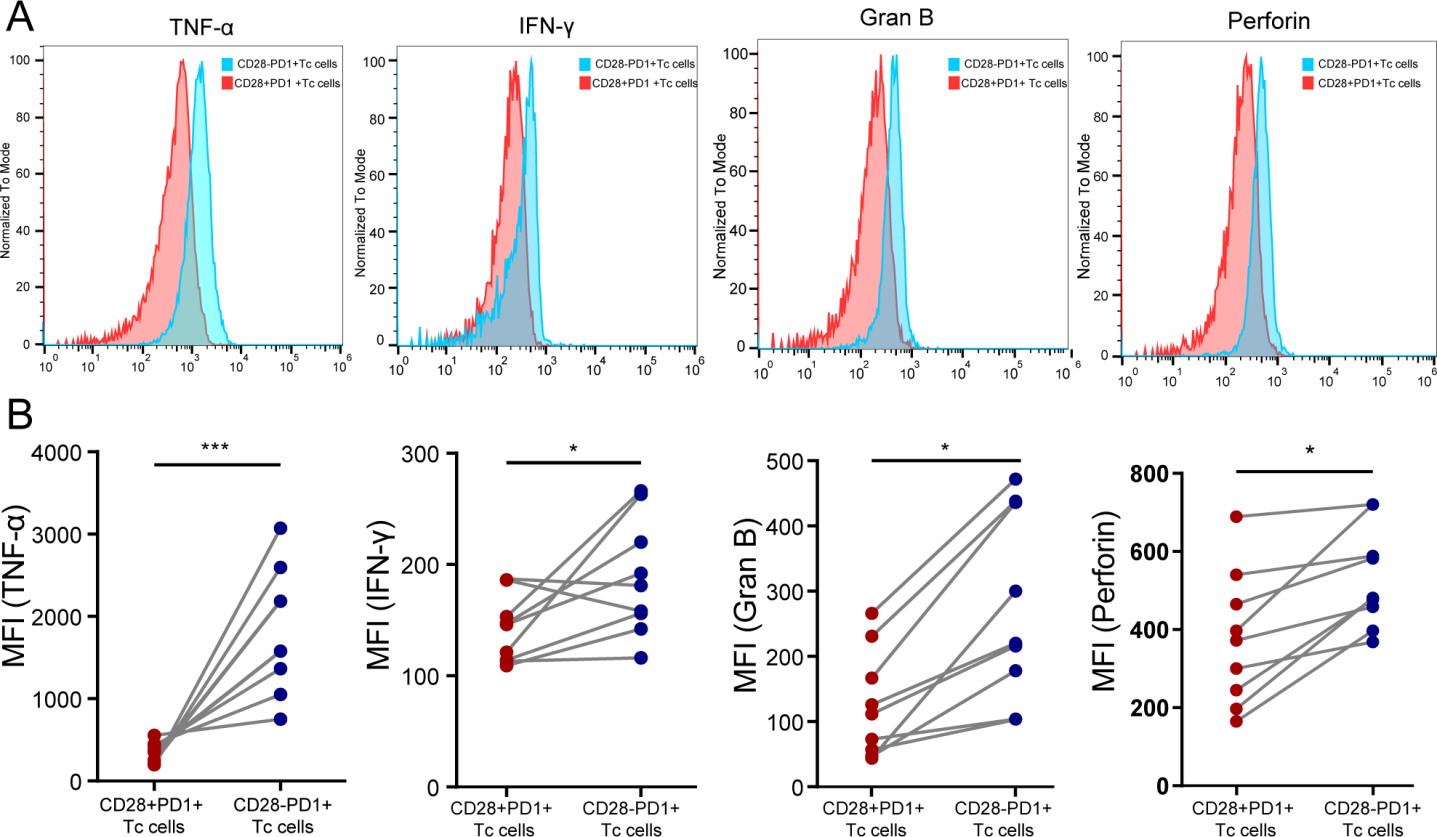
Assessment of the cytotoxic function of CD28^+^PD-1^+^ Tc cells in HCC tissues. **(A)** Representative distribution plot of TNF-α, IFN-γ, granzyme B, and perforin expression in CD28^+^PD-1^+^ Tc cells compared with CD28^-^PD-1^+^ Tc cells; **(B)** In HCC tissues, the expression levels of TNF-α (P<0.001), IFN-γ (P<0.05), granzyme B (P<0.05), and perforin (P<0.05) were significantly lower in CD28^+^PD-1^+^ Tc cells than in CD28-PD1+ Tc cells. HCC: hepatocellular carcinoma. *, p < 0.05; ***, p < 0.001.

We used flow cytometry to classify CD8^+^ T-cell subsets in HCC tissue and compared cytotoxic cytokine expression between CD28^+^PD-1^+^ Tc cells and CD28^-^PD-1^+^ Tc cells. The results showed that the expression levels of TNF-α, IFN-γ, granzyme B, and perforin were significantly lower in CD28^+^PD-1^+^ Tc cells than in CD28^-^PD-1^+^ Tc cells, which is consistent with the findings from the ELISA experiment (**Supplementary Figure S12A**), indicating impaired cytotoxic function and an exhausted state in CD28^+^PD-1^+^ Tc cells (**Figure 7**).

### 3.12 Decreased expression of immune checkpoints in CD28^+^PD-1^+^ Tc cells in HCC tissue

**Figure 8:**
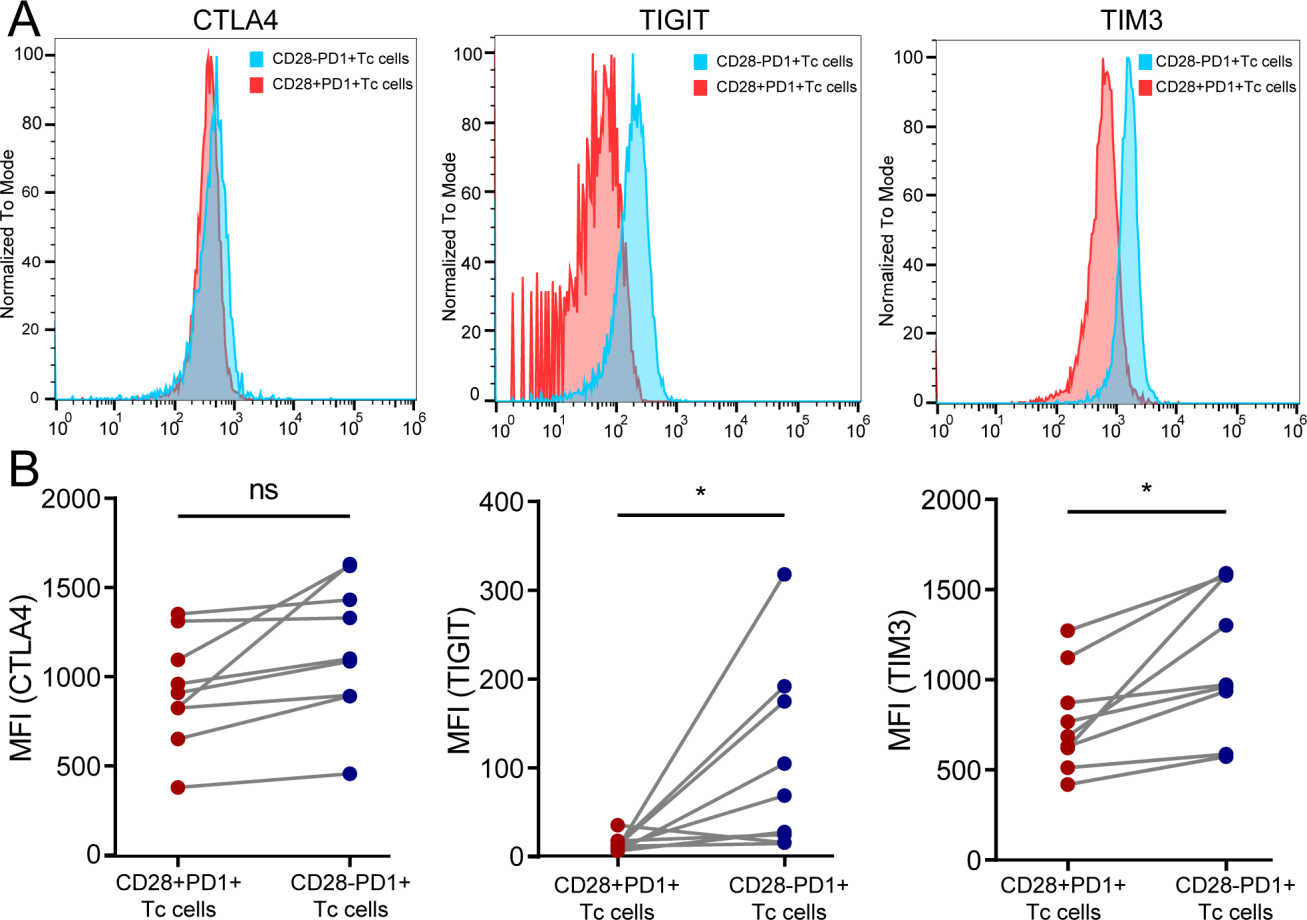
Detection of immune checkpoint molecule expression in CD28^+^PD-1^+^ Tc cells in HCC tissues. **(A)** Representative samples showing expression levels of CTLA4, TIGIT, and TIM3 in CD28^+^PD-1^+^ Tc cells compared with CD28^-^PD-1^+^ Tc cells; **(B)** In HCC tissues, the expression levels of TIGIT (p < 0.05) and TIM3 (p < 0.05) in CD28^+^PD-1^+^ Tc cells were significantly lower than those in CD28^-^PD1^+^ Tc cells, whereas CTLA4 expression did not show significant changes. HCC, hepatocellular carcinoma. ns, p ≥ 0.05; *, p < 0.05.

TIM3 and TIGIT expression levels were significantly lower in CD28^+^PD-1^+^ Tc cells than in CD28^-^PD-1^+^ T-cells in HCC tissue, whereas CTLA4 expression did not significantly change (**Figure 8**). This result is consistent with the RT-PCR findings (**Supplementary Figure S12B**). These findings indicated that the increased presence of CD28^+^PD-1^+^ Tc cells in HCC tissues may reduce immune therapy effectiveness owing to lower immune checkpoint molecule expression.

## 4 Discussion

HCC treatment has considerably progressed in recent years (37–39). However, HCC’s complex TME and high heterogeneity contribute to considerable variability in treatment responses among patients receiving the same therapy. Traditional tumor staging systems focus on static information, failing to capture dynamic changes in the TME and immune characteristics, which complicates accurate predictions of disease progression and treatment efficacy (40). Therefore, understanding the TME, identifying reliable biomarkers, and exploring novel therapeutic targets are necessary for the precise diagnosis and treatment of HCC (41).

Combining scRNA-seq with RNA-seq data permits a thorough analysis of the TME and immune response mechanisms, allowing the identification of particular T-cell subsets and enhancing the understanding of HCC’s TME (42, 43). Both scRNA-seq and flow cytometric analysis indicated a significant increase in the proportion of CD28^+^PD-1^+^ Tc cells within the HCC TME. Previous studies have demonstrated that although the overall number of CD8^+^ T cells is lower in non-small-cell lung cancer, the proportion of CD28^+^ PD-1^+^ Tc cells is notably higher in tumor tissues compared to peripheral blood and normal tissues, while the proportion of CD28^-^PD-1^+^ Tc cells remains unchanged. These findings are consistent with the results of the present study (15). CD28^+^PD-1^+^ Tc cells, as a key component in HCC, may play a crucial role. Further analysis revealed that CD28^+^PD-1^+^ Tc cells exhibit significantly lower expression of cytotoxic factors compared to CD28^-^PD-1^+^ Tc cells, suggesting a functional impairment in this CD8^+^ T cell subset. CD28 is an essential co-stimulatory molecule enhancing T-cell activation. The binding of CD28 to CD80 or CD86 on antigen-presenting cells activates T cells to generate positive signals (44). PD-1, part of the CD28 co-receptor family, blocks T-cell proliferation and activation by disrupting the PI3K/Akt signaling pathways associated with CD28 (45). Consequently, CD28 is a key target for PD-1 downstream inhibitory signaling, and T-cell reactivation after PD-1 blockade is critically dependent on CD28. The distinct expression patterns and interactions between CD28 and PD-1 are central to regulating T-cell function (46, 47). CD8^+^ T cells play a pivotal role in HCC’s TME and can exert strong immune responses when activated by cytokines or chemokines (48). However, persistent antigen stimulation resulting from chronic hepatitis and liver diseases may lead to continuous activation of CD28 and PD-1 signaling, resulting in an antigen tolerance response in HCC (49). This process contributes to CD8^+^ T-cell exhaustion in HCC, characterized by diminished proliferation, decreased cytokine secretion, and weakened cytotoxic activity (50). Therefore, we hypothesize that CD28^+^PD-1^+^ Tc cells in HCC may belong to an exhausted T-cell subset, significantly contributing to HCC progression.

Survival analysis revealed that patients with higher proportions of CD28^+^PD-1^+^ Tc cells had significantly shorter overall survival. Cox regression analysis identified the enrichment level of CD28^+^PD-1^+^ Tc cells as an independent prognostic factor for HCC. Even after adjusting for the impact of PD-1^+^ T cells, the independent prognostic association remained significant. These cells may exacerbate immune escape and tumor progression owing to their diminished cytotoxic function and immunosuppressive state, resulting in poorer patient outcomes. This finding further confirms the significant prognostic value of CD28^+^PD-1^+^ T cells in the prognosis of patients with HCC, providing new insights for clinical practice. Interestingly, in the Cox regression analysis, we found that sex was also an independent prognostic factor, with male patients showing significantly worse outcomes. This may be attributed to several factors. Firstly, from an epidemiological perspective, the incidence of HCC is significantly higher in men than in women, and this imbalance in sex distribution may further amplify the predictive role of sex in survival models (51). Secondly, sex differences play a key role in HCC. Studies have shown that male patients typically first present with more advanced tumor stages and worse prognosis at diagnosis, which may be related to higher hepatic iron deposition in men. This deposition could promote HCC progression and tumor resistance (52, 53). Our analysis indicates that sex as a variable had greater significance in the model than TNM staging. However, TNM staging is still included as an important variable, likely because the strong predictive power of CD28^+^PD-1^+^ Tc-cell levels may partially diminish the independent prognostic effects of clinical variables.

Cell-cell communication is significantly higher in tumor tissues than in normal tissues, which is critical for tumor formation and therapy resistance (54).HLA-E, a non-classical class I MHC molecule, is typically expressed at low levels in most human tissues but shows significant upregulation in HCC (55). HLA-E regulates natural killer (NK)-cell and T-cell functions through interactions with the NKG2A and NKG2C receptors (56, 57). Previous studies have shown that the CD94/NKG2/HLA-E signaling pathway negatively regulates NK-cell activity in HCC, which may contribute to tumor immune escape (58). γδ T cells play dual roles in the TME. They can inhibit HCC through signaling pathways, such as with the activation of NKG2D/DNAM-1, yet they can also upregulate PD-1 expression to suppress their own cytotoxic function, thus promoting tumor escape (59, 60). We observed significant upregulation of HLA-E expression on CD28^+^PD-1^+^ Tc cells in HCC, with enhanced interactions with the corresponding *NKG2C* receptors on γδ T cells. Although direct binding evidence is lacking, this finding suggests that CD28^+^PD-1^+^ Tc cells may regulate γδ T cells, potentially through *NKG2C*, by upregulating *HLA-E*, thereby exerting an immunosuppressive effect. CLEC2C is an early activation marker for CD4^+^ T cells that is rapidly expressed during immune responses. Elevated CLEC2C levels have been linked to enhanced immune function in HCC and correlate with improved patient outcomes (61). KLRB1, a marker of activated NK and T-cell subsets, modulates immune responses and is closely linked to tumorigenesis (62, 63). High KLRB1 expression in CD8^+^ T cells is associated with improved cytotoxicity and more robust proliferation, enhancing their tumor cell-killing ability (64, 65). In this study, the expression and interactions of CLEC2C in CD28^+^PD-1^+^ Tc cells and KLRB1 in CD4^+^ T cells were significantly reduced in HCC, consistent with previous literature. This underscores the importance of the CLEC2C signaling pathway in HCC antitumor immunity. This suggests that CD28^+^PD-1^+^ Tc cells may downregulate *CLEC2C* expression, thereby impacting *KLRB1* expression in CD4^+^ T cells and contributing to immune dysfunction within the HCC microenvironment.

Interestingly, enrichment analysis revealed a close association between CD28^+^PD-1^+^ Tc-cell enrichment and ribosomes. These findings suggested that CD28^+^PD-1^+^ Tc-cell function is closely linked to ribosomes and their protein synthesis processes. Ribosomes are highly conserved across evolutionary lines. Moreover, they are widely distributed throughout various tissues (66). Ribosomal protein expression is notably elevated in HCC (67). Certain ribosomal proteins, including RPL32 and RPL19, have been established as early diagnostic biomarkers for HCC and may serve as prognostic indicators (68). Studies have shown that mitochondrial ribosome defects lead to altered glucose metabolism and lactate accumulation, which exacerbate T-cell exhaustion and tumor infiltration, thereby accelerating HCC progression (69). Both CD28 and PD-1 modulate T-cell metabolism via the PI3K/Akt/mTOR signaling pathway, which also directly influences ribosomal synthesis and function (70). Therefore, CD28^+^PD-1^+^ Tc cells may influence ribosomal protein expression through PI3K/Akt/mTOR signaling pathway, with abnormal ribosomal protein levels further impairing cellular metabolism and worsening T-cell exhaustion. These findings contribute novel insights into the mechanisms through which CD28^+^PD-1^+^ Tc cells mediate immune evasion in HCC, highlighting the importance of ribosomal proteins in T-cell dysfunction.

To explore the molecular mechanisms and potential clinical significance of CD28^+^PD-1^+^ Tc cells in HCC, we conducted multidimensional analyses, including TMB, drug sensitivity prediction, and TIDE scoring, based on the enrichment level of CD28^+^PD-1^+^ Tc cells. In this study, *TP53* and *CTNNB1* exhibited high mutation frequencies in most HCC tissues, in line with earlier studies (71). *CTNNB1* mutant HCCs are typically better differentiated and have more favorable prognoses, whereas HCCs with TP53 mutations alone and without *CTNNB1* mutations tend to be more invasive (72). The TMB reflects the accumulation of somatic missense mutations and is often used as an indicator of genomic instability. A higher TMB can increase the number of neo-antigen sites, potentially enhancing T-cell recognition and activation and the antitumor immune response (73). Long-term stimulation by tumor-specific antigens derived from mutated genes in tumor cells may lead to T-cell exhaustion. However, our analysis showed no significant differences in TMB between patients with HCC with high and low CD28^+^PD-1^+^ Tc-cell enrichment, suggesting that the immune characteristics of this subset are driven more by phenotypic changes than by mutated genes.

TIGIT and TIM3 are important immune checkpoint molecules, and blocking their signaling pathways can effectively restore the function of T cells and NK cells, enhancing anti-tumor immune responses, making them potential therapeutic targets for HCC (74, 75). The expression of TIGIT and TIM3 on CD28^+^PD-1^+^ Tc cells significantly decreases, which is consistent with the finding that patients with high CD28^+^PD-1^+^ Tc-cell enrichment had significantly higher TIDE scores. This suggests that the high enrichment of CD28^+^PD-1^+^ Tc cells is associated with a strong tendency for immune escape and potentially lower benefits from immunotherapy. Interestingly, patients with HCC and high CD28^+^PD-1^+^ Tc-cell enrichment demonstrated increased sensitivity to common chemotherapy agents, such as vinorelbine, bortezomib, and docetaxel. Specifically, vinorelbine combined with sorafenib in HCC mouse models significantly inhibited tumor cell proliferation and angiogenesis, promoted tumor necrosis and apoptosis, and enhanced radiotherapy effects (76, 77). Bortezomib effectively inhibits HCC progression *in vitro* by activating the Hippo pathway (78). Docetaxel inhibits microtubule polymerization, preventing cell division, and may suppress tumor growth and extend survival in patients with HCC through apoptosis induction and TME modulation (79). This suggests that patients with high CD28^+^PD-1^+^ Tc-cell enrichment correspond to a TME with stronger immune suppression but greater sensitivity to chemotherapy. CD28^+^PD-1^+^ Tc-cell enrichment may serve as a potential marker in HCC, and therapeutic strategies targeting different levels of CD28^+^PD-1^+^ Tc-cell enrichment could offer new directions for improving HCC treatment outcomes.

To the best of our knowledge, this study is the first to identify the characteristics of the CD28^+^PD-1^+^ Tc-cell subset in HCC. This subset exhibited significant immune dysfunction, characterized by reduced cytotoxicity and lower immune checkpoint molecule expression, indicating CD8^+^ T-cell exhaustion. This study provides preliminary insights into the potential mechanisms and interactions of CD28^+^PD-1^+^ Tc cells in HCC, providing a novel perspective for understanding the TME in HCC and new approaches for predicting treatment responses and prognosis.

This study had some limitations. First, while we preliminarily explored the potential role of CD28^+^PD-1^+^ Tc cells in HCC, the molecular mechanisms within the PD-1^+^ T-cell subsets, as well as the specific regulatory role of CD28 signaling in T-cell exhaustion, still require further validation. Second, for the CD28^+^PD-1^+^ Tc-cell subset, we examined the expression levels of common cytokines and immune checkpoints using a limited sample, without including analysis of important immune checkpoints such as LAG-3. Future research will address this gap by expanding the comparison of immune regulatory molecules to define the specific immunological mechanisms of this cell subset. Additionally, we plan to increase the sample size to further strengthen the reliability of the results. Third, in drug sensitivity analysis, we utilized the Genomics of Drug Sensitivity in Cancer database; however, common standard treatments for HCC, such as sorafenib, have not yet been included, which affects the direct clinical translatability of our findings. In the future, we plan to integrate real-world clinical drug usage data or expand to other drug response databases (such as PRISM, CTRP) to comprehensively assess the response of key drugs in the context of CD28^+^PD-1^+^ Tc-cell enrichment, thus advancing the development of personalized treatment strategies.

## 5 Conclusion

Significant enrichment of CD28^+^PD-1^+^ Tc cells in HCC and their reduced immune function indicated cytotoxicity loss and impaired immune response, suggesting a state of exhaustion. CD28^+^PD1^+^ Tc cell enrichment may aid in stratifying patient prognosis and serve as a sensitive indicator for predicting treatment responses. These results offer crucial evidence for CD28^+^PD-1^+^ Tc-cell involvement in the TME, underscoring a potential treatment target for HCC.

## Data Availability

The original contributions presented in the study are included in the article/**Supplementary Material**. Further inquiries can be directed to the corresponding authors.

## References

[1] Siegel RL Miller KD Wagle NS Jemal A. Cancer statistics, 2023. CA Cancer J Clin. (2023) 73:17–48. doi: 10.3322/caac.21763 36633525

[2] Li Q Xia C Li H Yan X Yang F Cao M. Disparities in 36 cancers across 185 countries: secondary analysis of global cancer statistics. Front Med. (2024) 18:911–20. doi: 10.1007/s11684-024-1058-6 39167345

[3] Dagogo-Jack I Shaw AT. Tumour heterogeneity and resistance to cancer therapies. Nat Rev Clin Oncol. (2018) 15:81–94. doi: 10.1038/nrclinonc.2017.166 29115304

[4] Desa DE Strawderman RL Wu W Hill RL Smid M Martens J. Intratumoral heterogeneity of second-harmonic generation scattering from tumor collagen and its effects on metastatic risk prediction. BMC Cancer. (2020) 20:1217. doi: 10.1186/s12885-020-07713-4 33302909 PMC7731482

[5] El-Kenawi A Hanggi K Ruffell B. The immune microenvironment and cancer metastasis. Cold Spring Harb Perspect Med. (2020) 10:a037424. doi: 10.1101/cshperspect.a037424 31501262 PMC7117953

[6] Jansen CS Prokhnevska N Master VA Sanda MG Carlisle JW Bilen MA. An intra-tumoral niche maintains and differentiates stem-like CD8 T cells. Nature. (2019) 576:465–70. doi: 10.1038/s41586-019-1836-5 PMC7108171 31827286

[7] Philip M Schietinger A. CD8(+) T cell differentiation and dysfunction in cancer. Nat Rev Immunol. (2022) 22:209–23. doi: 10.1038/s41577-021-00574-3 PMC9792152 34253904

[8] Xiao S Lu L Lin Z Ye X Su S Zhang C. LAYN serves as a prognostic biomarker and downregulates tumor-infiltrating CD8(+) T cell function in hepatocellular carcinoma. J Hepatocell Carcinoma. (2024) 11:1031–48. doi: 10.2147/JHC.S464806 PMC11164088 38859944

[9] Humblin E Korpas I Lu J Filipescu D van der Heide V Goldstein S. Sustained CD28 costimulation is required for self-renewal and differentiation of TCF-1(+) PD-1(+) CD8 T cells. Sci Immunol. (2023) 8:eadg0878. doi: 10.1126/sciimmunol.adg0878 37624910 PMC10805182

[10] Milner JJ Toma C He Z Kurd NS Nguyen QP McDonald B. Heterogenous populations of tissue-resident CD8(+) T cells are generated in response to infection and Malignancy. Immunity. (2020) 52:808–24. doi: 10.1016/j.immuni.2020.04.007 PMC7784612 32433949

[11] Konjar S Ficht X Iannacone M Veldhoen M. Heterogeneity of tissue resident memory T cells. Immunol Lett. (2022) 245:1–07. doi: 10.1016/j.imlet.2022.02.009 35346744

[12] Klein GR O’Sullivan D Corrado M Bremser A Buck MD Buescher JM. Mitochondrial priming by CD28. Cell. (2017) 171:385–97. doi: 10.1016/j.cell.2017.08.018 PMC5637396 28919076

[13] Marinelli O Annibali D Aguzzi C Tuyaerts S Amant F Morelli MB. The controversial role of PD-1 and its ligands in gynecological Malignancies. Front Oncol. (2019) 9:1073. doi: 10.3389/fonc.2019.01073 31681606 PMC6803534

[14] Hui E Cheung J Zhu J Su X Taylor MJ Wallweber HA. T cell costimulatory receptor CD28 is a primary target for PD-1-mediated inhibition. Science. (2017) 355:1428–33. doi: 10.1126/science.aaf1292 PMC6286077 28280247

[15] Palermo B Franzese O Frisullo G D’Ambrosio L Panetta M Campo G. CD28/PD1 co-expression: dual impact on CD8(+) T cells in peripheral blood and tumor tissue, and its significance in NSCLC patients’ survival and ICB response. J Exp Clin Cancer Res. (2023) 42:287. doi: 10.1186/s13046-023-02846-3 37898752 PMC10612243

[16] Colaprico A Silva TC Olsen C Garofano L Cava C Garolini D. TCGAbiolinks: an R/Bioconductor package for integrative analysis of TCGA data. Nucleic Acids Res. (2016) 44:e71. doi: 10.1093/nar/gkv1507 26704973 PMC4856967

[17] Butler A Hoffman P Smibert P Papalexi E Satija R. Integrating single-cell transcriptomic data across different conditions, technologies, and species. Nat Biotechnol. (2018) 36:411–20. doi: 10.1038/nbt.4096 PMC6700744 29608179

[18] Qiu X Mao Q Tang Y Wang L Chawla R Pliner HA. Reversed graph embedding resolves complex single-cell trajectories. Nat Methods. (2017) 14:979–82. doi: 10.1038/nmeth.4402 PMC5764547 28825705

[19] Karmaus P Chen X Lim SA Herrada AA Nguyen TM Xu B. Metabolic heterogeneity underlies reciprocal fates of T(H)17 cell stemness and plasticity. Nature. (2019) 565:101–05. doi: 10.1038/s41586-018-0806-7 PMC6420879 30568299

[20] Fang Z Tian Y Sui C Guo Y Hu X Lai Y. Single-cell transcriptomics of proliferative phase endometrium: systems analysis of cell-cell communication network using cellChat. Front Cell Dev Biol. (2022) 10:919731. doi: 10.3389/fcell.2022.919731 35938159 PMC9352955

[21] Blake JA Christie KR Dolan ME Drabkin HJ Hill DP Ni L. Gene Ontology Consortium: going forward. Nucleic Acids Res. (2015) 43:D1049–56. doi: 10.1093/nar/gku1179 PMC4383973 25428369

[22] Kanehisa M Goto S. KEGG: kyoto encyclopedia of genes and genomes. Nucleic Acids Res. (2000) 28:27–30. doi: 10.1093/nar/28.1.27 10592173 PMC102409

[23] Yu G Wang LG Han Y He QY. clusterProfiler: an R package for comparing biological themes among gene clusters. Omics. (2012) 16:284–87. doi: 10.1089/omi.2011.0118 PMC3339379 22455463

[24] Wu S Lv X Li Y Gao X Ma Z Fu X. Integrated machine learning and single-sample gene set enrichment analysis identifies a TGF-beta signaling pathway derived score in headneck squamous cell carcinoma. J Oncol. (2022) 2022:3140263. doi: 10.1155/2022/3140263 36090900 PMC9458367

[25] National Comprehensive Cancer Network (NCCN). NCCN clinical practice guidelines in oncology (NCCN Guidelines^®^) for geriatrics version 1.2023. National Comprehensive Cancer Network (2023).

[26] Ritchie ME Phipson B Wu D Hu Y Law CW Shi W. limma powers differential expression analyses for RNA-sequencing and microarray studies. Nucleic Acids Res. (2015) 43:e47. doi: 10.1093/nar/gkv007 25605792 PMC4402510

[27] Subramanian A Tamayo P Mootha VK Mukherjee S Ebert BL Gillette MA. Gene set enrichment analysis: a knowledge-based approach for interpreting genome-wide expression profiles. Proc Natl Acad Sci U.S.A. (2005) 102:15545–50. doi: 10.1073/pnas.0506580102 PMC1239896 16199517

[28] Liberzon A Birger C Thorvaldsdottir H Ghandi M Mesirov JP Tamayo P. The Molecular Signatures Database (MSigDB) hallmark gene set collection. Cell Syst. (2015) 1:417–25. doi: 10.1016/j.cels.2015.12.004 PMC4707969 26771021

[29] Liberzon A Subramanian A Pinchback R Thorvaldsdottir H Tamayo P Mesirov JP. Molecular signatures database (MSigDB) 3.0. Bioinformatics. (2011) 27:1739–40. doi: 10.1093/bioinformatics/btr260 PMC3106198 21546393

[30] Ru B Wong CN Tong Y Zhong JY Zhong S Wu WC. TISIDB: an integrated repository portal for tumor-immune system interactions. Bioinformatics. (2019) 35:4200–02. doi: 10.1093/bioinformatics/btz210 30903160

[31] Ito K Murphy D. Application of ggplot2 to pharmacometric graphics. Cpt Pharmacometrics Syst Pharmacol. (2013) 2:e79. doi: 10.1038/psp.2013.56 24132163 PMC3817376

[32] Yang W Soares J Greninger P Edelman EJ Lightfoot H Forbes S. Genomics of Drug Sensitivity in Cancer (GDSC): a resource for therapeutic biomarker discovery in cancer cells. Nucleic Acids Res. (2013) 41:D955–61. doi: 10.1093/nar/gks1111 PMC3531057 23180760

[33] Maeser D Gruener RF Huang RS. oncoPredict: an R package for predicting *in vivo* or cancer patient drug response and biomarkers from cell line screening data. Brief Bioinform. (2021) 22:bbab260. doi: 10.1093/bib/bbab260 34260682 PMC8574972

[34] Mayakonda A Lin DC Assenov Y Plass C Koeffler HP. Maftools: efficient and comprehensive analysis of somatic variants in cancer. Genome Res. (2018) 28:1747–56. doi: 10.1101/gr.239244.118 PMC6211645 30341162

[35] Liu Z Wang L Guo C Liu L Jiao D Sun Z. TTN/OBSCN ‘Double-Hit’ predicts favourable prognosis, ‘immune-hot’ subtype and potentially better immunotherapeutic efficacy in colorectal cancer. J Cell Mol Med. (2021) 25:3239–51. doi: 10.1111/jcmm.16393 PMC8034451 33624434

[36] Brinkman EK Chen T Amendola M van Steensel B. Easy quantitative assessment of genome editing by sequence trace decomposition. Nucleic Acids Res. (2014) 42:e168. doi: 10.1093/nar/gku936 25300484 PMC4267669

[37] Chi H Zhao S Yang J Gao X Peng G Zhang J. T-cell exhaustion signatures characterize the immune landscape and predict HCC prognosis via integrating single-cell RNA-seq and bulk RNA-sequencing. Front Immunol. (2023) 14:1137025. doi: 10.3389/fimmu.2023.1137025 37006257 PMC10050519

[38] Miller KD Nogueira L Devasia T Mariotto AB Yabroff KR Jemal A. Cancer treatment and survivorship statistics, 2022. CA Cancer J Clin. (2022) 72:409–36. doi: 10.3322/caac.21731 35736631

[39] Wei L Owen D Rosen B Guo X Cuneo K Lawrence TS. A deep survival interpretable radiomics model of hepatocellular carcinoma patients. Phys Med. (2021) 82:295–305. doi: 10.1016/j.ejmp.2021.02.013 33714190 PMC8035300

[40] Che YQ Zhang Y Li HB Shen D Cui W. Serum KLKB1 as a potential prognostic biomarker for hepatocellular carcinoma based on data-independent acquisition and parallel reaction monitoring. J Hepatocell Carcinoma. (2021) 8:1241–52. doi: 10.2147/JHC.S325629 PMC8520450 34676182

[41] He Q Yang J Jin Y. Immune infiltration and clinical significance analyses of the coagulation-related genes in hepatocellular carcinoma. Brief Bioinform. (2022) 23:bbac291. doi: 10.1093/bib/bbac291 35849048

[42] Zhang Y Wang D Peng M Tang L Ouyang J Xiong F. Single-cell RNA sequencing in cancer research. J Exp Clin Cancer Res. (2021) 40:81. doi: 10.1186/s13046-021-01874-1 33648534 PMC7919320

[43] Qu C Yan X Wei Y Tang F Li Y. Establishment and validation of a novel CD8+ T cell-associated prognostic signature for predicting clinical outcomes and immunotherapy response in hepatocellular carcinoma via integrating single-cell RNA-seq and bulk RNA-seq. Discovery Oncol. (2024) 15:235. doi: 10.1007/s12672-024-01092-z PMC11190115 38900330

[44] Hsu PN Yang TC Kao JT Cheng KS Lee YJ Wang YM. Increased PD-1 and decreased CD28 expression in chronic hepatitis B patients with advanced hepatocellular carcinoma. Liver Int. (2010) 30:1379–86. doi: 10.1111/j.1478-3231.2010.02323.x 20738778

[45] Gianchecchi E Delfino DV Fierabracci A. Recent insights into the role of the PD-1/PD-L1 pathway in immunological tolerance and autoimmunity. Autoimmun Rev. (2013) 12:1091–100. doi: 10.1016/j.autrev.2013.05.003 23792703

[46] Duraiswamy J Turrini R Minasyan A Barras D Crespo I Grimm AJ. Myeloid antigen-presenting cell niches sustain antitumor T cells and license PD-1 blockade via CD28 costimulation. Cancer Cell. (2021) 39:1623–42. doi: 10.1016/j.ccell.2021.10.008 PMC8861565 34739845

[47] Kim KH Kim HK Kim HD Kim CG Lee H Han JW. PD-1 blockade-unresponsive human tumor-infiltrating CD8(+) T cells are marked by loss of CD28 expression and rescued by IL-15. Cell Mol Immunol. (2021) 18:385–97. doi: 10.1038/s41423-020-0427-6 PMC8027446 32332901

[48] Wherry EJ Kurachi M. Molecular and cellular insights into T cell exhaustion. Nat Rev Immunol. (2015) 15:486–99. doi: 10.1038/nri3862 PMC4889009 26205583

[49] Ringelhan M Pfister D O’Connor T Pikarsky E Heikenwalder M. The immunology of hepatocellular carcinoma. Nat Immunol. (2018) 19:222–32. doi: 10.1038/s41590-018-0044-z 29379119

[50] Seimiya T Otsuka M Fujishiro M. Overcoming T-cell exhaustion: New therapeutic targets in HCC immunotherapy. Hepatology. (2023) 78:1009–11. doi: 10.1097/HEP.0000000000000039 36974835

[51] Radu IP Scheiner B Schropp J Delgado MG Schwacha-Eipper B Jin C. The influence of sex and age on survival in patients with hepatocellular carcinoma. Cancers (Basel). (2024) 16:4023. doi: 10.3390/cancers16234023 39682209 PMC11640092

[52] Nishida N Arizumi T Hayaishi S Takita M Kitai S Yada N. Gender differences in the livers of patients with hepatocellular carcinoma and chronic hepatitis C infection. Dig Dis. (2012) 30:547–53. doi: 10.1159/000343057 23258093

[53] Chen J Wang X Ye W. Prognostic analysis of sex and age in hepatocellular carcinoma: a SEER study. Eur J Gastroenterol Hepatol. (2024) 36:646–51. doi: 10.1097/MEG.0000000000002745 PMC10965129 38555602

[54] Meacham CE Morrison SJ. Tumour heterogeneity and cancer cell plasticity. Nature. (2013) 501:328–37. doi: 10.1038/nature12624 PMC4521623 24048065

[55] Wang XK Liao XW Yang CK Yu TD Liu ZQ Gong YZ. Diagnostic and prognostic biomarkers of Human Leukocyte Antigen complex for hepatitis B virus-related hepatocellular carcinoma. J Cancer. (2019) 10:5173–90. doi: 10.7150/jca.29655 PMC6775598 31602270

[56] Kaiser BK Barahmand-Pour F Paulsene W Medley S Geraghty DE Strong RK. Interactions between NKG2x immunoreceptors and HLA-E ligands display overlapping affinities and thermodynamics. J Immunol. (2005) 174:2878–84. doi: 10.4049/jimmunol.174.5.2878 15728498

[57] Kaiser BK Pizarro JC Kerns J Strong RK. Structural basis for NKG2A/CD94 recognition of HLA-E. Proc Natl Acad Sci U.S.A. (2008) 105:6696–701. doi: 10.1073/pnas.0802736105 PMC2373352 18448674

[58] Wu M Mei F Liu W Jiang J. Comprehensive characterization of tumor infiltrating natural killer cells and clinical significance in hepatocellular carcinoma based on gene expression profiles. BioMed Pharmacother. (2020) 121:109637. doi: 10.1016/j.biopha.2019.109637 31810126

[59] Jiang H Yang Z Song Z Green M Song H Shao Q. gammadelta T cells in hepatocellular carcinoma patients present cytotoxic activity but are reduced in potency due to IL-2 and IL-21 pathways. Int Immunopharmacol. (2019) 70:167–73. doi: 10.1016/j.intimp.2019.02.019 30802679

[60] Yin K Chu K Li M Duan Y Yu Y Kang M. Immune regulatory networks and therapy of gammadelta T cells in liver cancer: recent trends and advancements. J Clin Transl Hepatol. (2024) 12:287–97. doi: 10.1038/s41598-023-34261-1 PMC10899867 38426194

[61] Tang K Li X Mo J Chen Y Huang C Li T. CD69 serves as a potential diagnostic and prognostic biomarker for hepatocellular carcinoma. Sci Rep. (2023) 13:7452. doi: 10.1038/s41598-023-34261-1 37156819 PMC10167346

[62] Zhang Z Bahabayi A Liu D Hasimu A Zhang Y Guo S. KLRB1 defines an activated phenotype of CD4+ T cells and shows significant upregulation in patients with primary Sjogren’s syndrome. Int Immunopharmacol. (2024) 133:112072. doi: 10.1016/j.intimp.2024.112072 38636371

[63] Braud VM Meghraoui-Kheddar A Elaldi R Petti L Germain C Anjuere F. LLT1-CD161 interaction in cancer: promises and challenges. Front Immunol. (2022) 13:847576. doi: 10.3389/fimmu.2022.847576 35185935 PMC8854185

[64] Li Z Zheng B Qiu X Wu R Wu T Yang S. The identification and functional analysis of CD8+PD-1+CD161+ T cells in hepatocellular carcinoma. NPJ Precis Oncol. (2020) 4:28. doi: 10.1038/s41698-020-00133-4 33145436 PMC7599220

[65] Fang S Zhou Y. Deciphering the role of KLRB1: a novel prognostic indicator in hepatocellular carcinoma. BMC Gastroenterol. (2024) 24:210. doi: 10.1186/s12876-024-03299-4 38914941 PMC11194965

[66] Bowman JC Petrov AS Frenkel-Pinter M Penev PI Williams LD. Root of the tree: the significance, evolution, and origins of the ribosome. Chem Rev. (2020) 120:4848–78. doi: 10.1021/acs.chemrev.9b00742 32374986

[67] El KW Nasr Z. Deregulation of ribosomal proteins in human cancers. Biosci Rep. (2021) 41:BSR20211577. doi: 10.1042/BSR20211577 34873618 PMC8685657

[68] Hou G Lu Z Jiang J Yang X. Ribosomal protein L32 enhances hepatocellular carcinoma progression. Cancer Med. (2023) 12:10791–803. doi: 10.1002/cam4.5811 PMC10225200 37017565

[69] Hannan KM Sanij E Hein N Hannan RD Pearson RB. Signaling to the ribosome in cancer–It is more than just mTORC1. IUBMB Life. (2011) 63:79–85. doi: 10.1002/iub.428 21360636

[70] Wang S Liu C Yang C Jin Y Cui Q Wang D. PI3K/AKT/mTOR and PD-1/CTLA-4/CD28 pathways as key targets of cancer immunotherapy (Review). Oncol Lett. (2024) 28:567. doi: 10.3892/ol.2024.14700 39390982 PMC11465225

[71] Zucman-Rossi J Villanueva A Nault JC Llovet JM. Genetic landscape and biomarkers of hepatocellular carcinoma. Gastroenterology. (2015) 149:1226–39. doi: 10.1053/j.gastro.2015.05.061 26099527

[72] Nishida N Nishimura T Kaido T Minaga K Yamao K Kamata K. Molecular scoring of hepatocellular carcinoma for predicting metastatic recurrence and requirements of systemic chemotherapy. Cancers (Basel). (2018) 10:367. doi: 10.3390/cancers10100367 30274313 PMC6210853

[73] Innocenti F Ou FS Qu X Zemla TJ Niedzwiecki D Tam R. Mutational analysis of patients with colorectal cancer in CALGB/SWOG 80405 identifies new roles of microsatellite instability and tumor mutational burden for patient outcome. J Clin Oncol. (2019) 37:1217–27. doi: 10.1200/JCO.18.01798 PMC6506418 30865548

[74] Ledderose S Ledderose C Ledderose GJ. Expression of immune checkpoint molecules TIGIT and TIM-3 by tumor-infiltrating lymphocytes predicts poor outcome in sinonasal mucosal melanoma. Pathol Res Pract. (2024) 260:155468. doi: 10.1016/j.prp.2024.155468 39018929

[75] Lu X Liu J Cui P Liu T Piao C Xu X. Co-inhibition of TIGIT, PD1, and Tim3 reverses dysfunction of Wilms tumor protein-1 (WT1)-specific CD8+ T lymphocytes after dendritic cell vaccination in gastric cancer. Am J Cancer Res. (2018) 8:1564–75. PMC6129483 30210924

[76] Ng WH Soo KC Huynh H. Vinorelbine improves the efficacy of sorafenib against hepatocellular carcinoma: A promising therapeutic approach. Int J Mol Sci. (2024) 25:1563. doi: 10.3390/ijms25031563 38338842 PMC10855313

[77] Yeoh KW Prawira A Saad M Lee KM Lee E Low GK. Vinorelbine augments radiotherapy in hepatocellular carcinoma. Cancers (Basel). (2020) 12:872. doi: 10.3390/cancers12040872 32260169 PMC7226273

[78] Liao Y Hu K Liu W Wang W Qiu H Pan S. Bortezomib inhibits hepatocellular carcinoma via the Hippo-Yes-associated protein signalling pathway. Basic Clin Pharmacol Toxicol. (2023) 132:297–311. doi: 10.1111/bcpt.13832 36585038

[79] Liu YC Su CW Ko PS Lee RC Liu CJ Huang YH. A clinical trial with valproic acid and hydralazine in combination with gemcitabine and cisplatin followed by doxorubicin and dacarbazine for advanced hepatocellular carcinoma. Asia Pac J Clin Oncol. (2022) 18:19–27. doi: 10.1111/ajco.13443 32964588

