# Supplementary material for: Role of CD28+ PD-1+ Tc cells in immune response and prognosis prediction in hepatocellular carcinoma
Source: Front Immunol. 2025 Jun 4;16:1576193. doi: 10.3389/fimmu.2025.1576193 (PMC12174045; doi:10.3389/fimmu.2025.1576193)
Supplement: Supplementary file 14 [file Table4.docx]

Supplementary Material
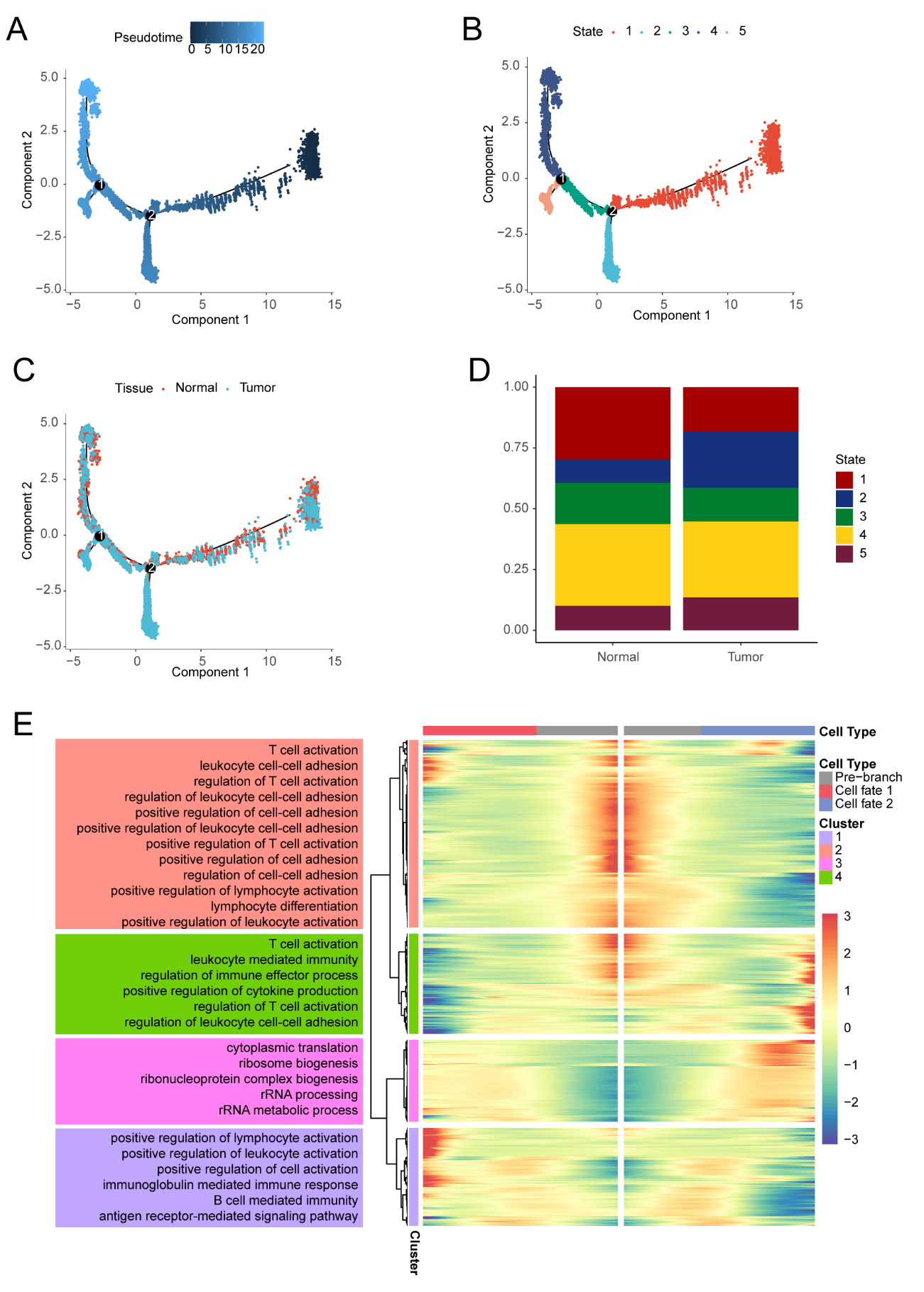


**Supplementary Figure 1** Pseudotime analysis reveals T-cell transcription patterns. **(A)** Pseudotime color gradient transitioning from deep blue to light blue. **(B)** Displays the pseudotime trajectory segmented into five distinct states using Monocle2. **(C)** Pseudotime trajectory showing cell distribution for HCC and control groups based on grouping. **(D)** Stacked bar chart displaying the distribution of different states in HCC and normal tissues. **(E)** Heatmap showing DEGs across different T-cell fates, with significantly enriched GO pathways for various gene clusters displayed on the left. HCC: hepatocellular carcinoma; DEGs: differentially expressed genes; GO: Gene Ontology

**
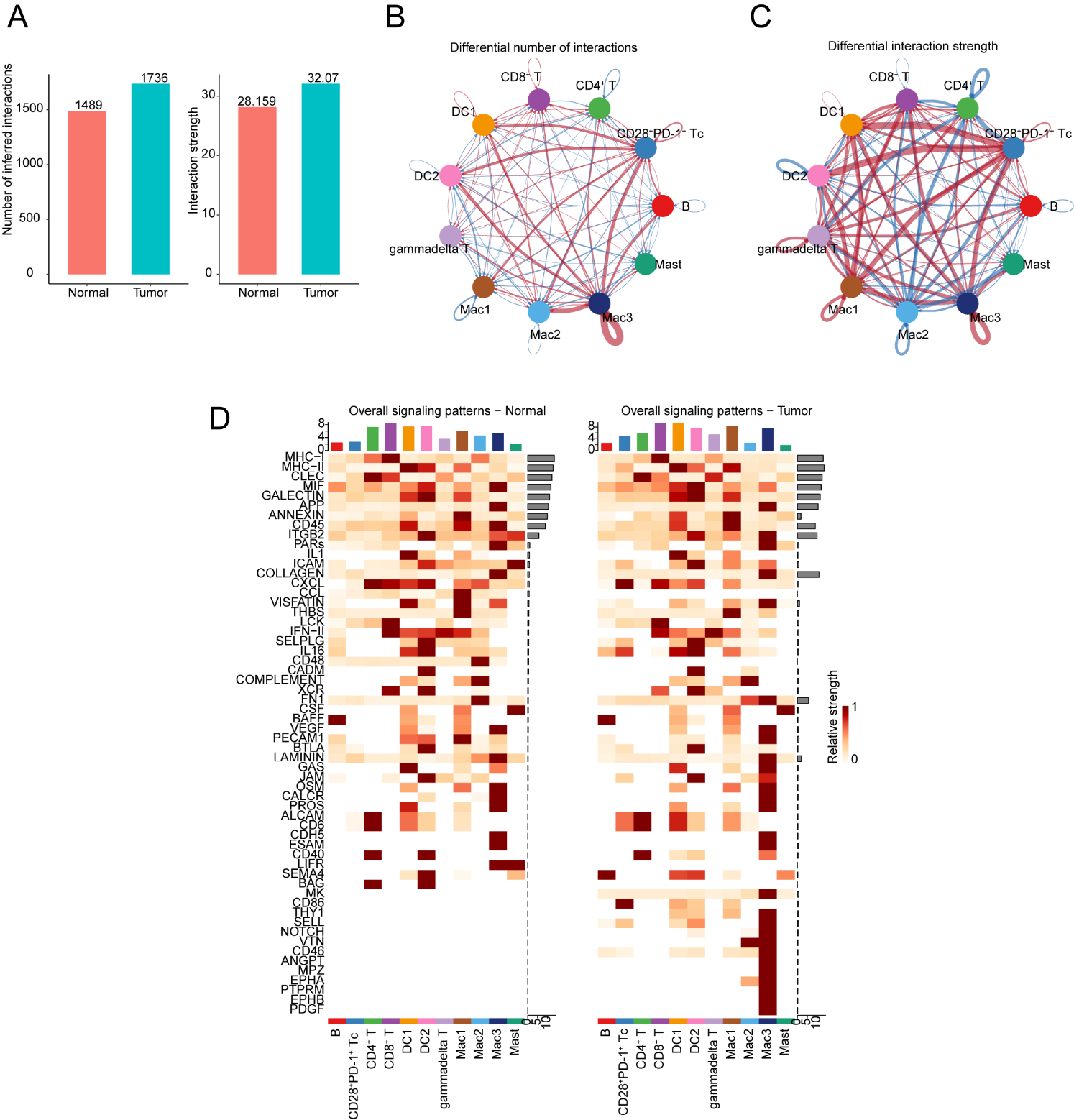
Supplementary Figure 2** Analysis of intercellular communication. **(A)** Bar chart illustrating the number and intensity of interactions between normal and HCC cell types. **(B)** Network diagram depicting the volume of interactions among cells. **(C)** Network diagram illustrating the strength of interactions among cells. **(D)** Heatmap displaying the signaling pathways that play the largest role in the overall signaling in HCC and normal tissues. HCC: hepatocellular carcinoma

**
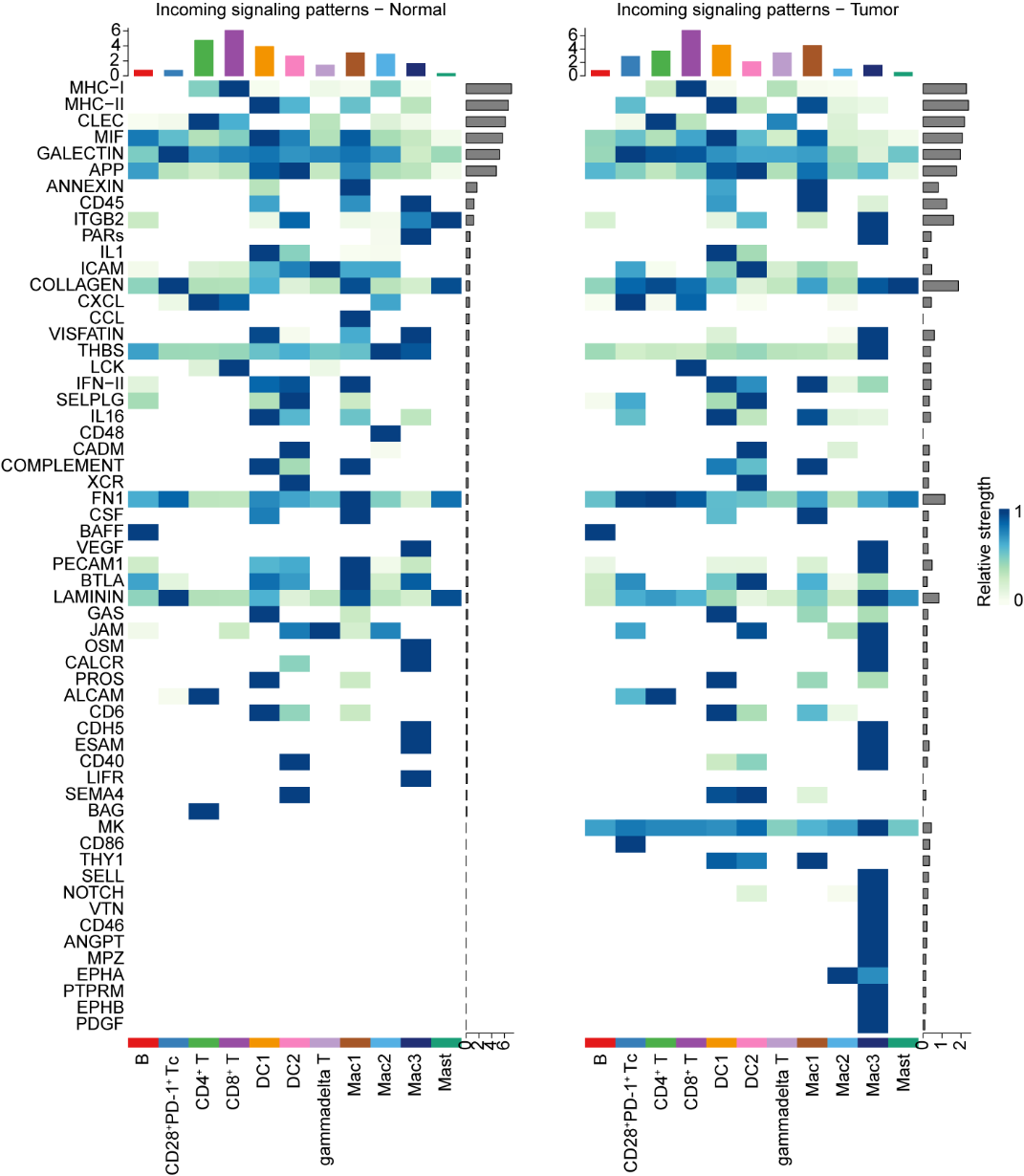
**

**Supplementary Figure 3** Heatmap depicting the signaling pathways of cell types in HCC and normal tissues. HCC: hepatocellular carcinoma


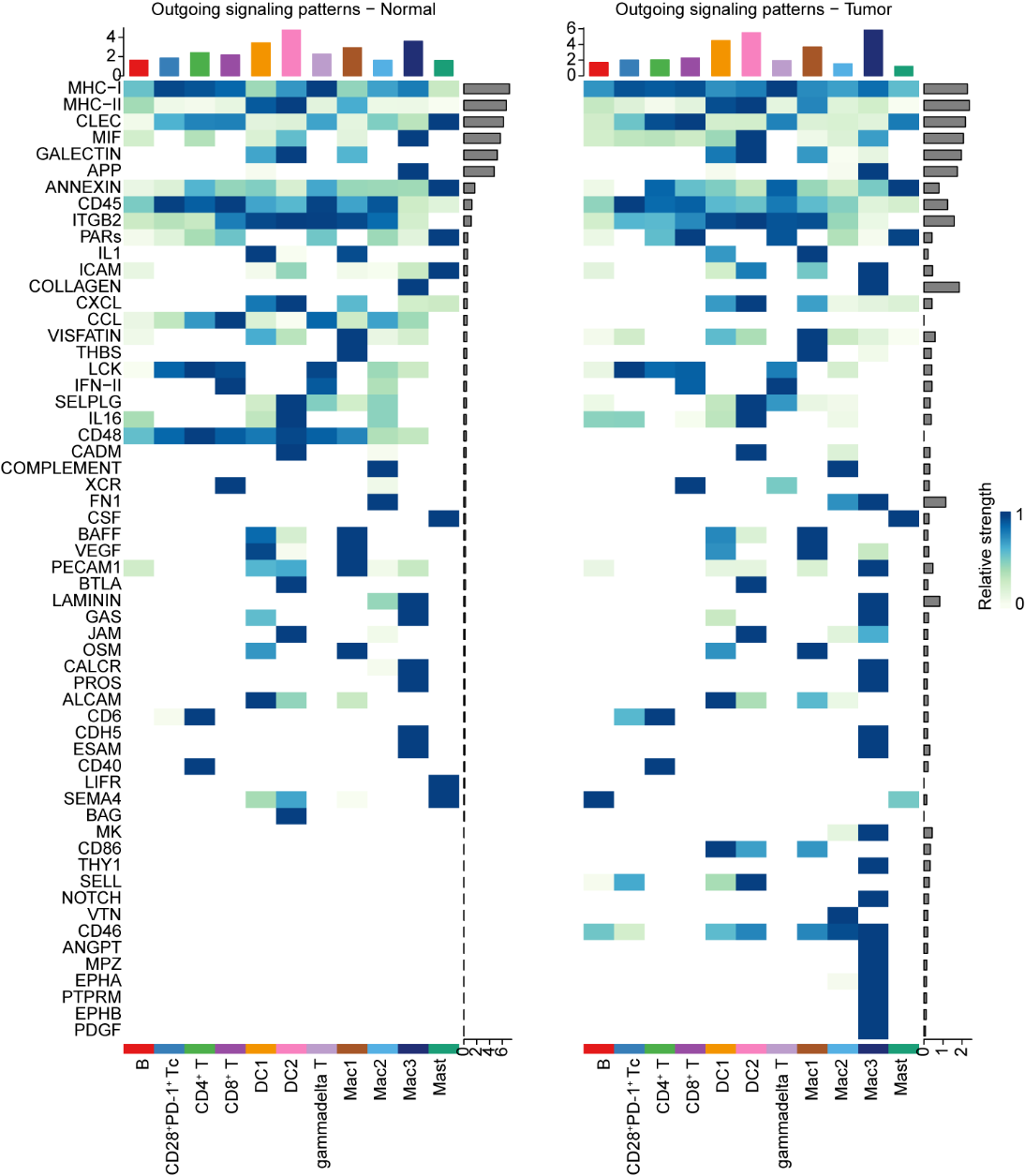


**Supplementary Figure 4** Heatmap illustrating the signaling pathways of cell types in HCC and normal tissues. HCC: hepatocellular carcinoma


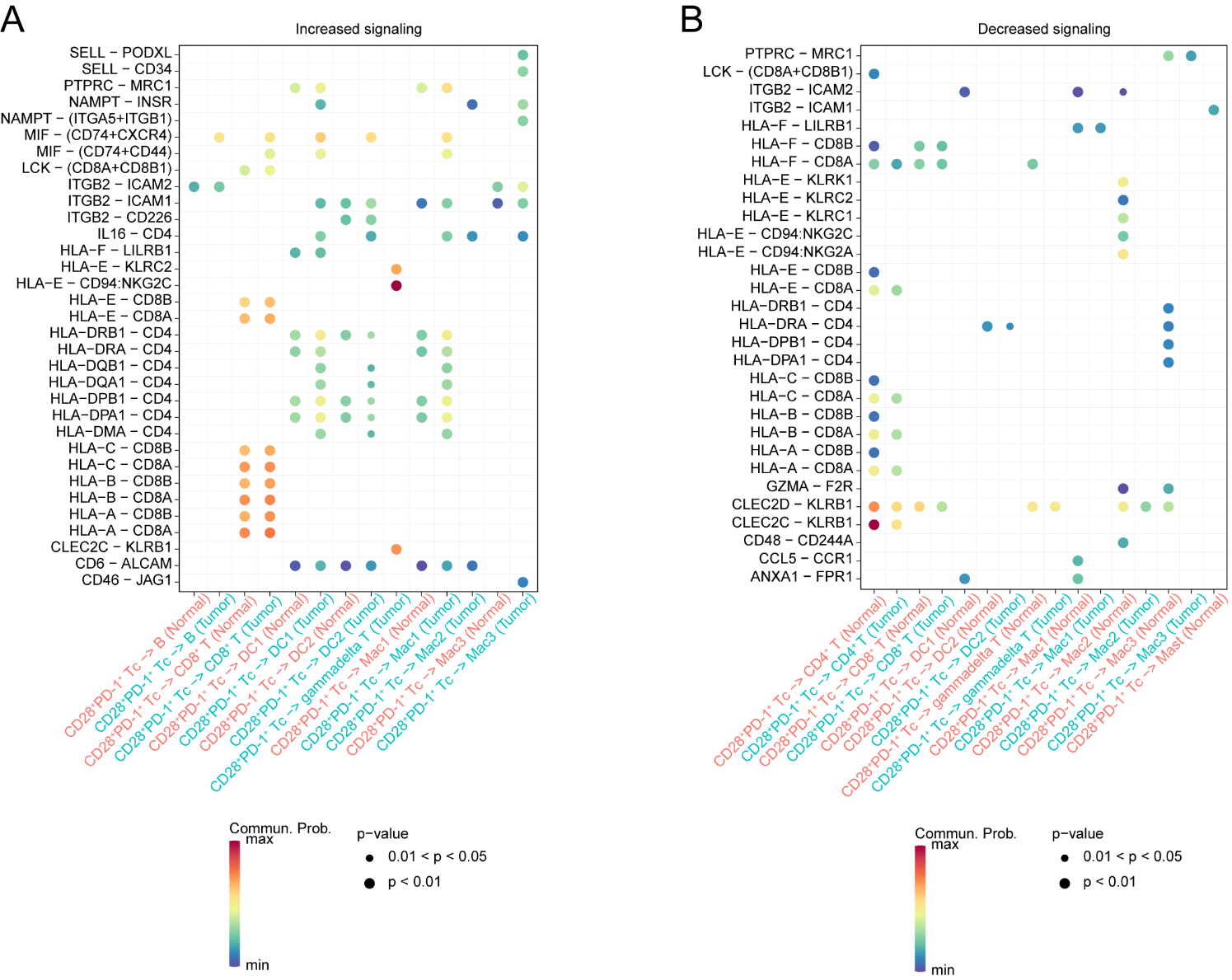


**Supplementary Figure 5** Signals emitted by CD28+PD-1+ T-cells in normal and HCC cell communication. **(A)** The communication intensity of CD28+PD-1+ T-cells with other cell subsets increased for certain receptors in HCC. **(B)** The communication intensity of CD28+PD-1+ T-cells with other cell subpopulations decreased for certain receptors in HCC. HCC: hepatocellular carcinoma


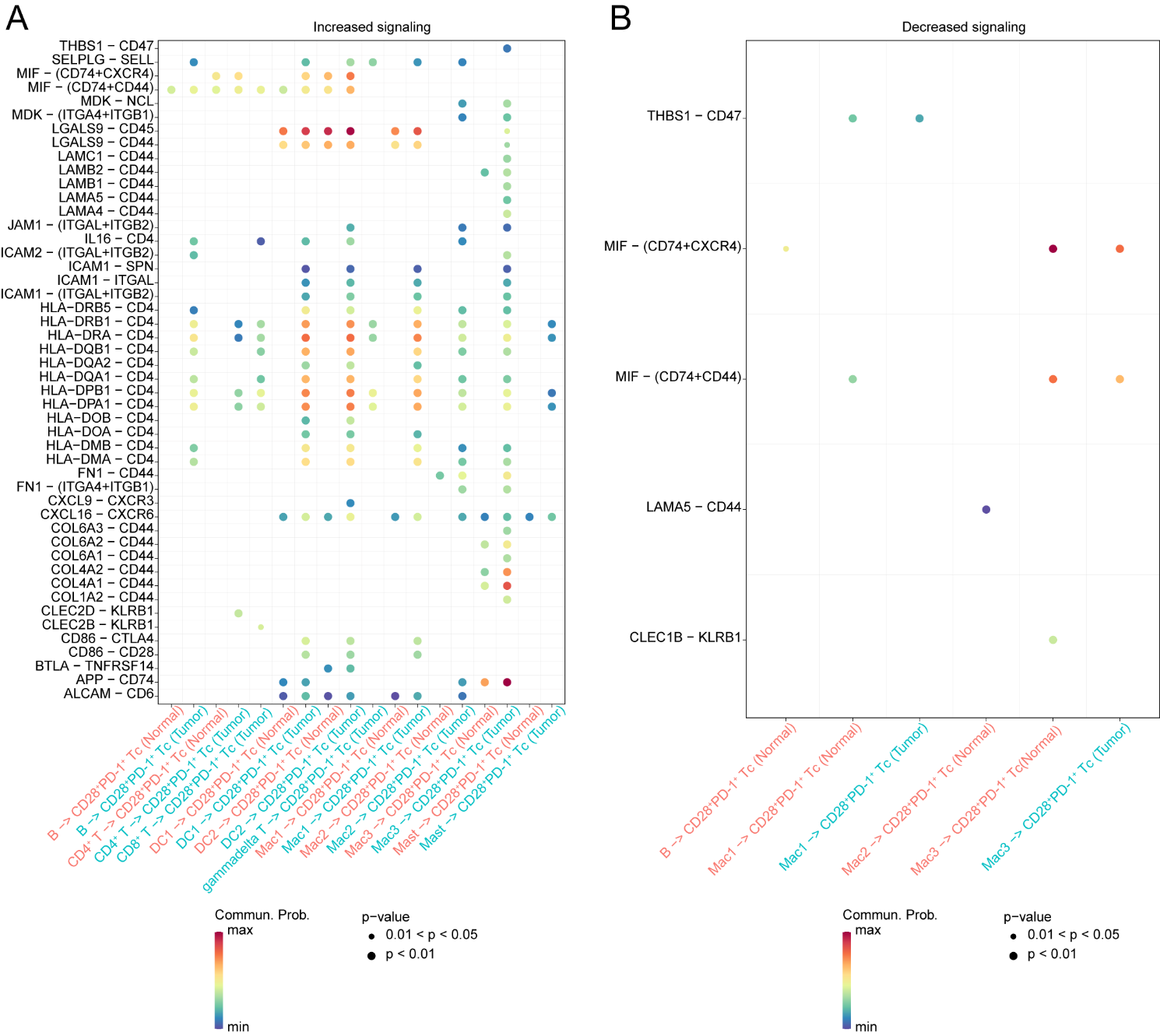


**Supplementary Figure 6** Signals received by CD28+PD-1+T-cells in normal and HCC cell communication. **(A)** The communication intensity of CD28+PD-1+ T-cells with other cell subpopulations increased for certain receptors in HCC. **(B)** The communication intensity of CD28+PD-1+ T-cells with other cell subpopulations decreased for certain receptors in HCC. HCC: hepatocellular carcinoma


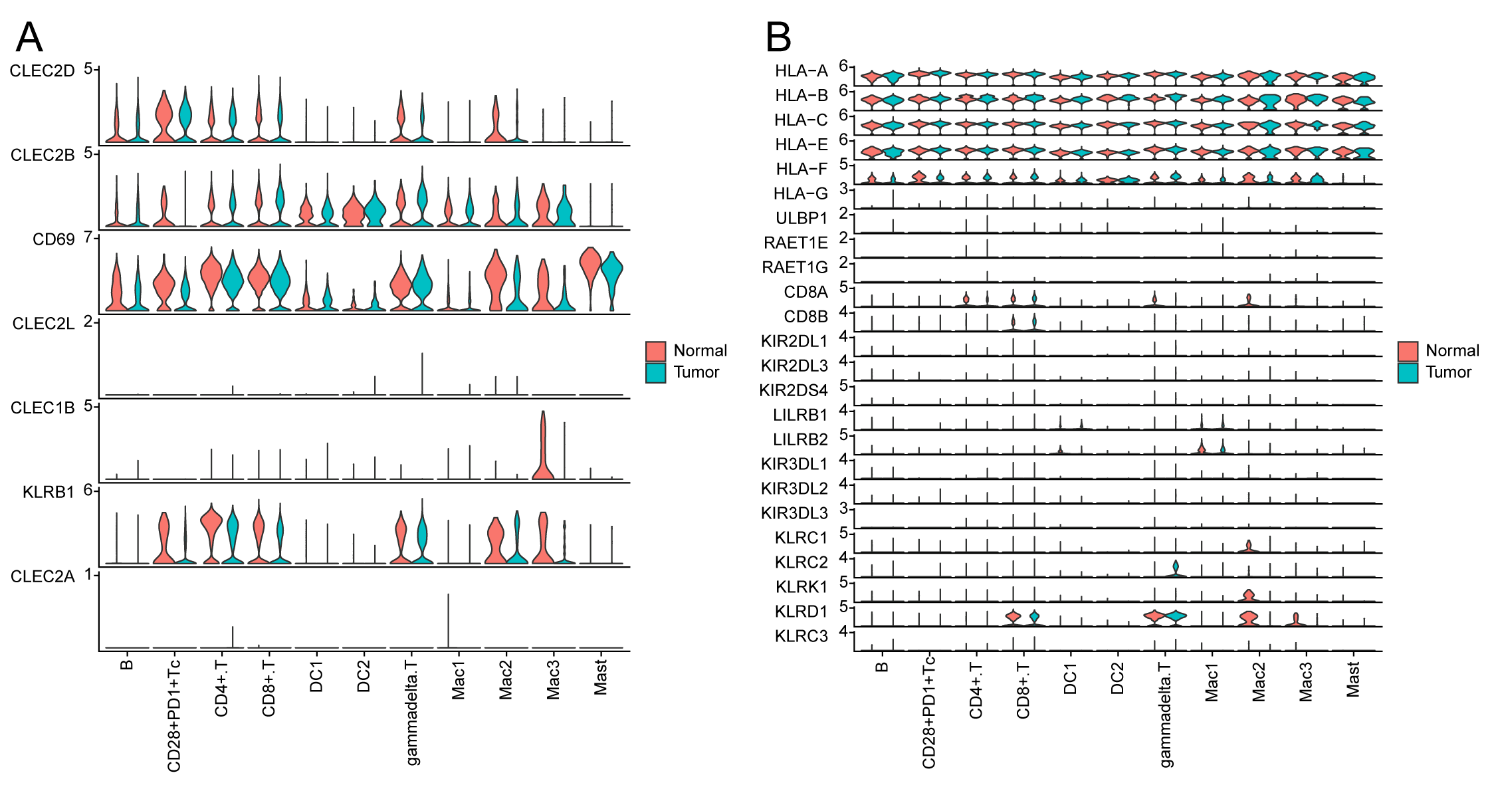


**Supplementary Figure 7** Receptor expression for CLEC2C and HLA-E signaling pathways in normal cells and HCC. **(A)** Distribution of CLEC2C signaling receptor expression in HCC compared to normal groups. **(B)** Distribution of HLA-E signaling receptor expression in HCC and normal groups. HCC: hepatocellular carcinoma


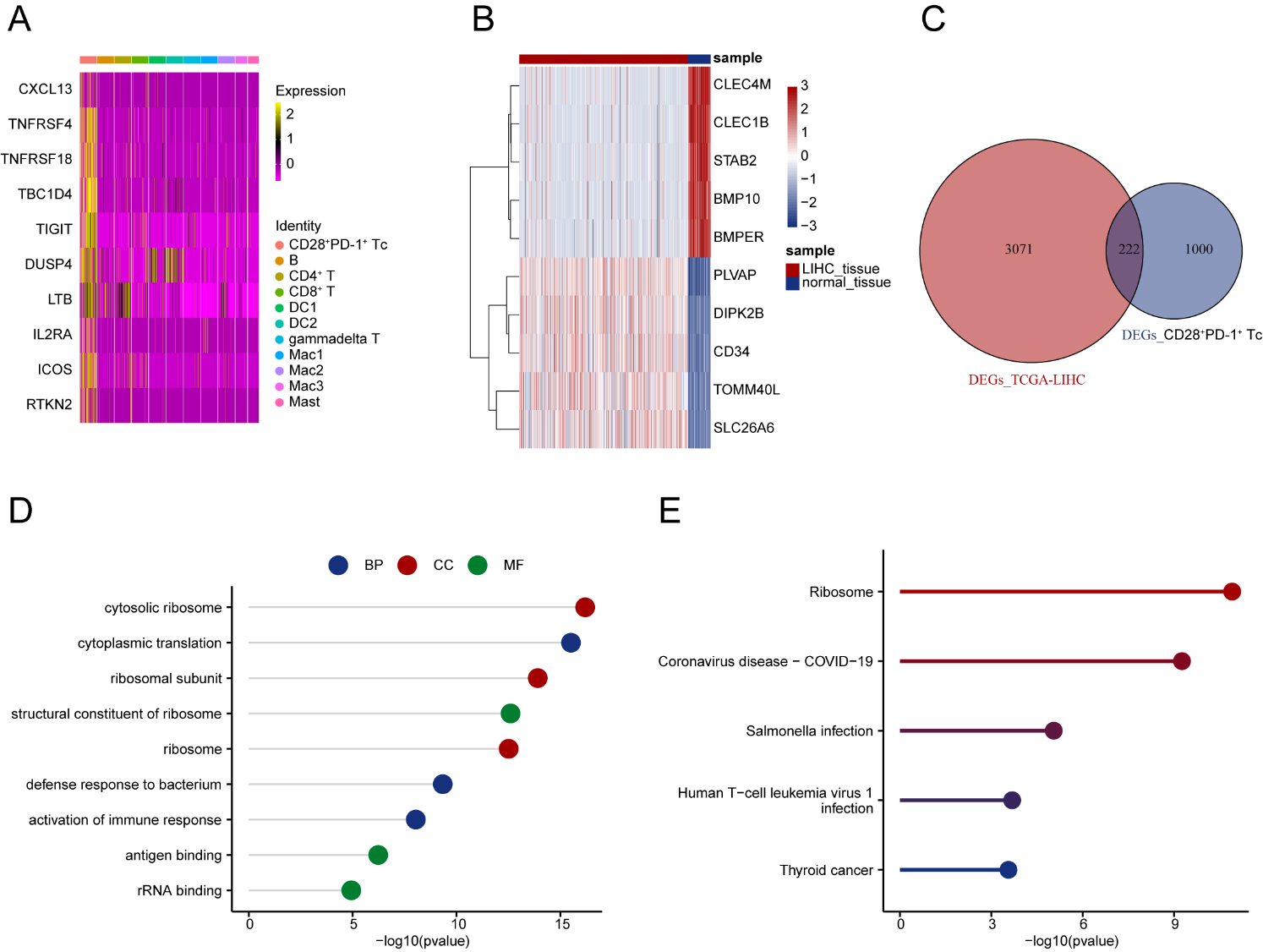


**Supplementary Figure 8** Enrichment analysis of DEGs associated with CD28+PD-1+ T-cells in HCC. **(A)** Heatmap showing 10 significantly upregulated DEGs in CD28+PD-1+ T-cells. **(B)** Heatmap displaying 10 significantly downregulated DEGs. **(C)** Venn diagram highlighting hub genes. **(D)** GO analysis of hub genes. **(E)** KEGG analysis of hub genes. HCC: hepatocellular carcinoma; DEGs: differentially expressed genes; GO: Gene Ontology; KEGG: Kyoto Encyclopedia of Genes and Genomes


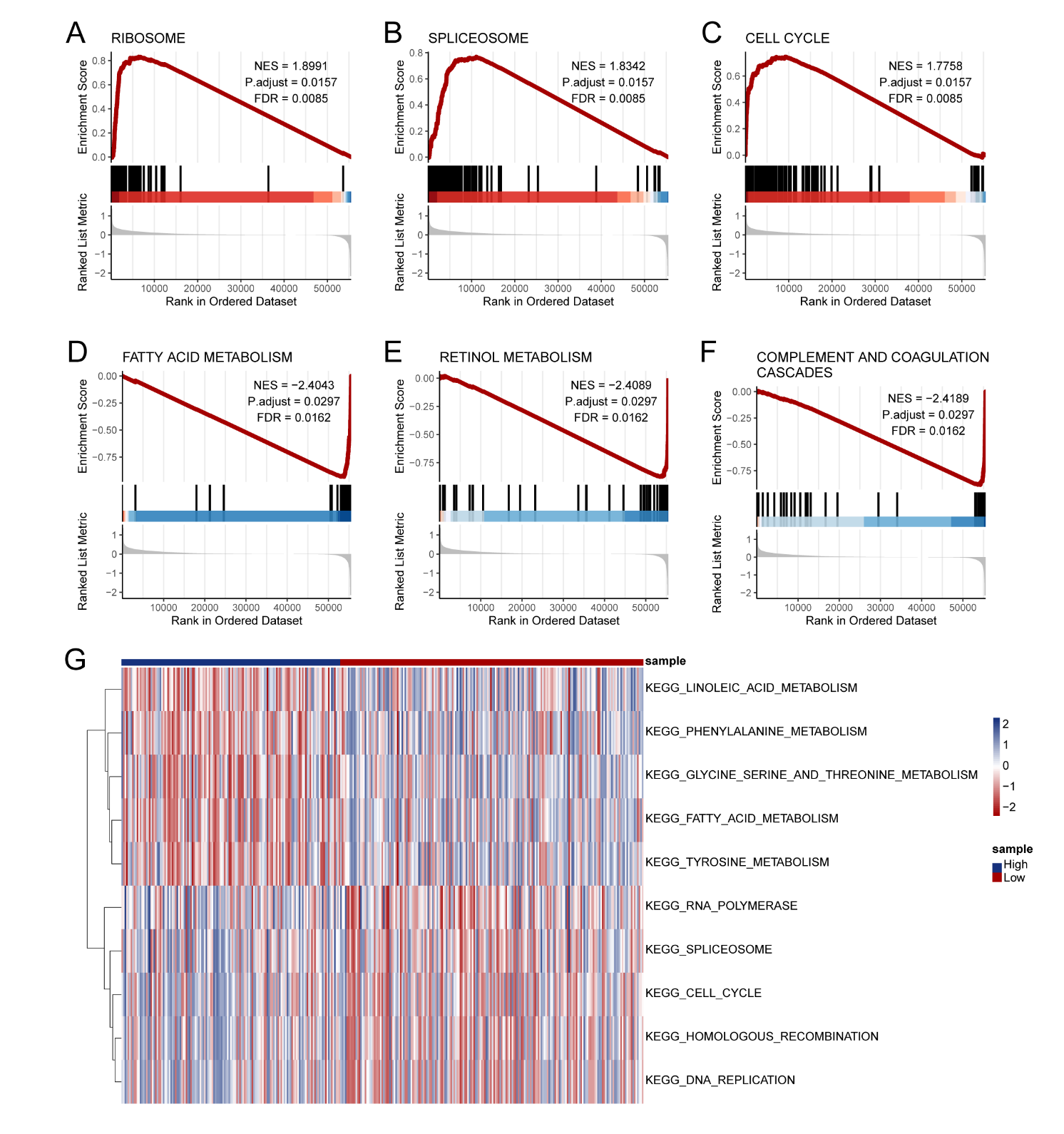


**Supplementary Figure 9** Significantly enriched pathways from GSEA and GSVA. GSEA indicates significant enrichment of pathways such as RIBOSOME **(A),** SPLICEOSOME **(B)**, CELL CYCLE **(C)**, FATTY ACID METABOLISM **(D)**, RETINOL METABOLISM **(E)**, and COMPLEMENT AND COAGULATION CASCADES **(F)**. **(G)** Heatmap of pathways differentially enriched between high- and low-risk groups from GSVA. NES: normalized enrichment score; GSVA: gene set variation analysis; GSEA: gene set enrichment analysis

**
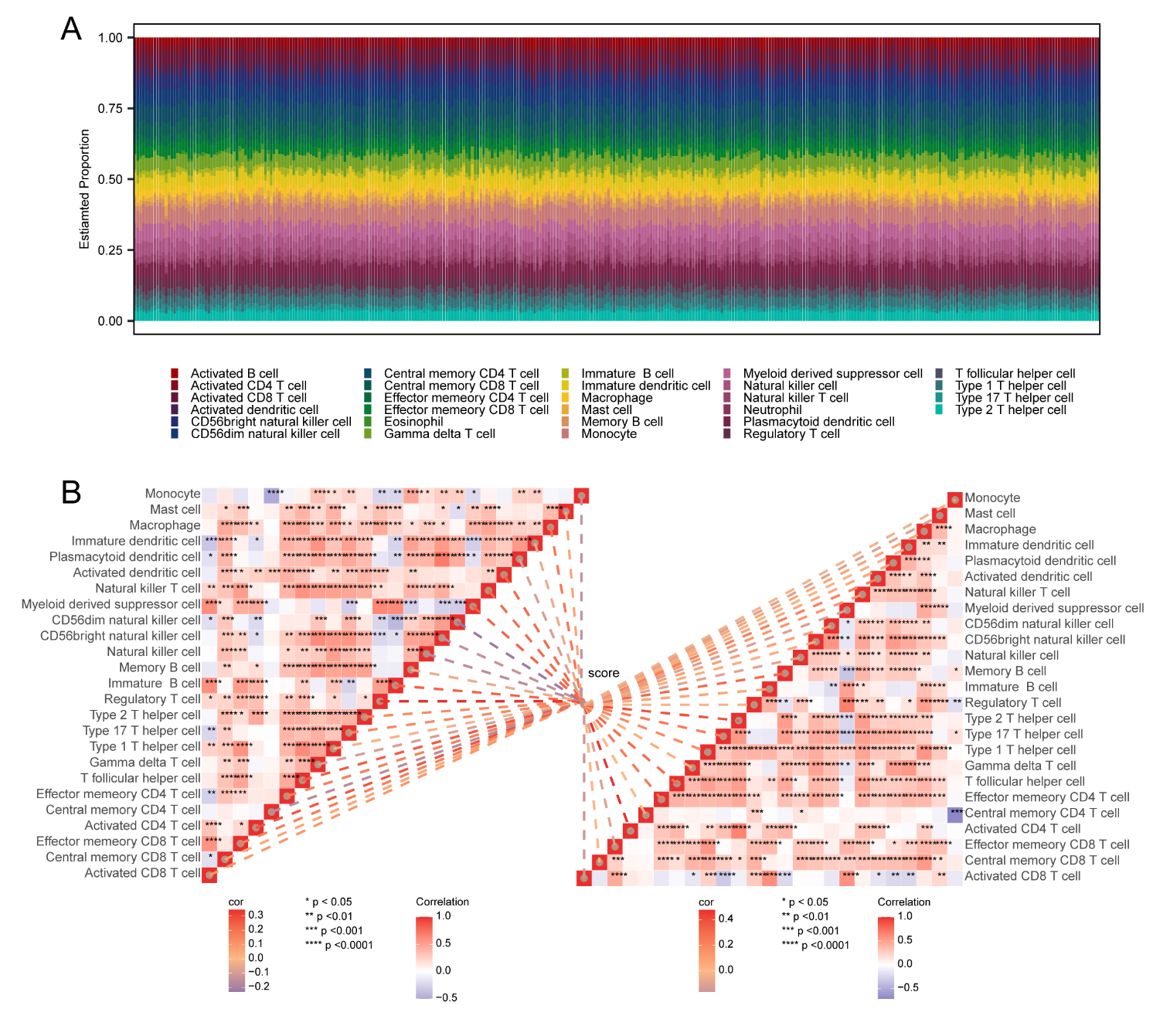
**

**Supplementary Figure 10** Comparison of immune infiltration levels between high- and low-enrichment groups. **(A)** Immune infiltration landscape in HCC depicted through a histogram of relative immune cell proportions across all samples. **(B)** Correlation among immune cells. HCC: hepatocellular carcinoma


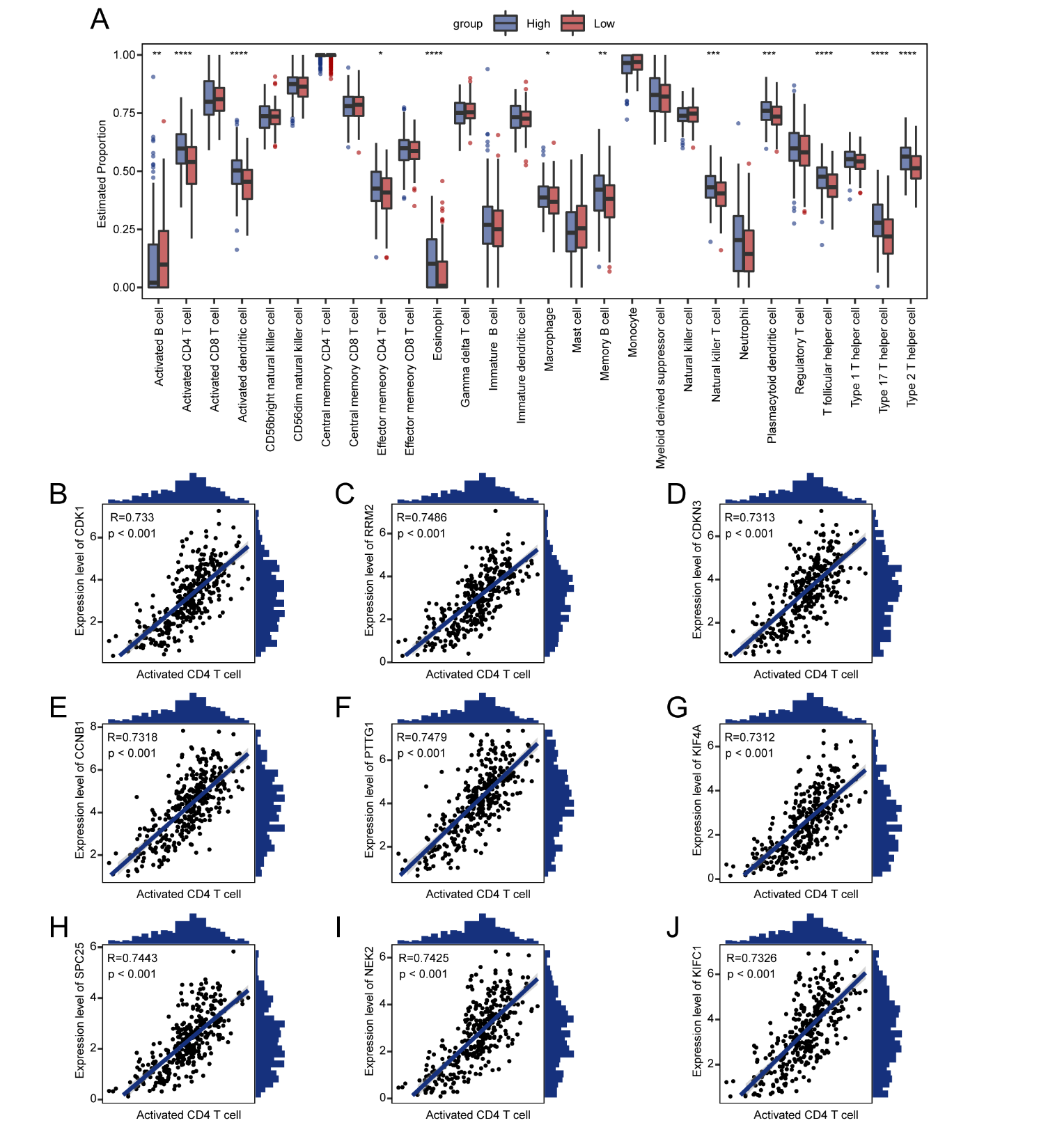


**Supplementary Figure 11** Correlation scatter plots. **(A)** Boxplot showing the estimated proportions of immune cells in groups with high and low enrichment. Correlation of activated CD4+ T-cells with genes CDK1 **(B),** RRM2 **(C)**, CDKN3 **(D)**, CCNB1 **(E)**, PTTG1 **(F)**, KIF4A **(G)**, SPC25 **(H)**, NEK2 **(I),** and KIFC1 **(J)** in relation to activated CD4 T-cell immune infiltration.


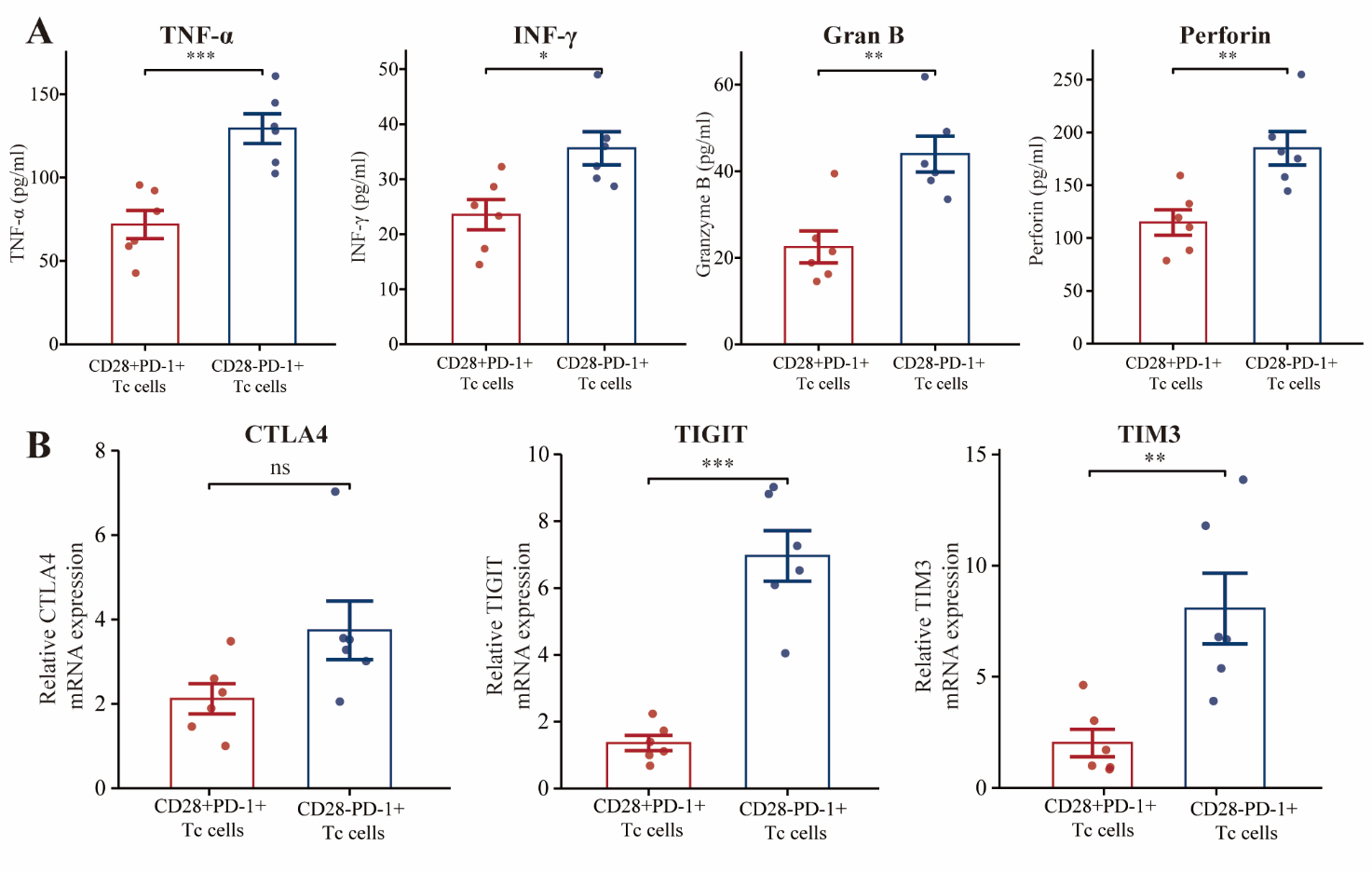


**Supplementary Figure 12** Assessment of the cytotoxic function and immune checkpoint molecule expression in CD28^+^PD-1^+^ Tc cells within HCC tissues. **(A)** ELISA analysis revealed that the expression levels of cytotoxic molecules TNF-α, IFN-γ, granzyme B, and perforin were significantly lower in CD28^+^PD-1^+^ Tc cells compared to CD28^-^PD-1^+^ Tc cells from HCC tissues (all *p* < 0.05). **(B)** rt-PCR analysis indicated that the expression of immune checkpoint molecules TIGIT and TIM3 was significantly reduced in CD28^+^PD-1^+^ Tc cells compared to CD28^-^PD-1^+^ Tc cells (both *p* < 0.05), while CTLA4 expression showed no significant difference between these subsets. HCC: hepatocellular carcinoma.
