# Supplementary material for: Role of CD28+ PD-1+ Tc cells in immune response and prognosis prediction in hepatocellular carcinoma
Source: Front Immunol. 2025 Jun 4;16:1576193. doi: 10.3389/fimmu.2025.1576193 (PMC12174045; doi:10.3389/fimmu.2025.1576193)
Supplement: Supplementary file 15 [file Table5.docx]

Antibodies Details

| Antibodies | Company | Country | Catalog number | Clone | Fluorochrome |
| --- | --- | --- | --- | --- | --- |
| TNF-α | Biolegend | USA | 502913 | MAb11 | APC |
| IFN-γ | Biolegend | USA | 502511 | 4S.B3 | APC |
| Granzyme B | Biolegend | USA | 396408 | QA18A28 | APC |
| Perforin | Biolegend | USA | 353311 | B-D48 | APC |
| CD152 (CTLA-4) | Biolegend | USA | 349907 | L3D10 | APC |
| TIGIT | Biolegend | USA | 372705 | A15153G | APC |
| TIM-3 | Biolegend | USA | 364803 | A18087E | APC |
| CD8 | eBioscience | USA | 17-0088-42 | RPA-T8 | APC |
| CD28 | eBioscience | USA | 12-0289-42 | CD28.2 | PE |
| PD1 | eBioscience | USA | 11-9969-42 | MIH4 | FITC |

Microbeads Details

| Names | Company | Country | Catalog number |
| --- | --- | --- | --- |
| CD3 MicroBeadKit | Miltenyi | Germany | 30-097-043 |
| CD8 MicroBeadKit | Miltenyi | Germany | 130-045-201 |

Gene primer sequences

| Gene | Forward primer | Reverse primer |
| --- | --- | --- |
| GAPDH | GTCTCCTCTGACTTCAACAGCG | ACCACCCTGTTGCTGTAGCCAA |
| TIGIT | TGGTGGTCATCTGCACAGCAGT | TTTCTCCTGAGGTCACCTTCCAC |
| TIM-3 | GACTCTAGCAGACAGTGGGATC | GGTGGTAAGCATCCTTGGAAAGG |
| CTLA4 | ACGGGACTCTACATCTGCAAGG | GGAGGAAGTCAGAATCTGGGCA |

GAPDH was used as an internal control gene.

Elisa Kit Details

| Names | Company | Country | Catalog number |
| --- | --- | --- | --- |
| Human Granzyme B Elisa Kit | WEIAOBIO | China | EH10206S |
| Human IFN-γ Elisa Kit | WEIAOBIO | China | EH10230S |
| Human Perforin ELISA kit | WEIAOBIO | China | EH10391S |
| Human TNF-α Elisa Kit | WEIAOBIO | China | EH10497S |

Gating strategies for HCC


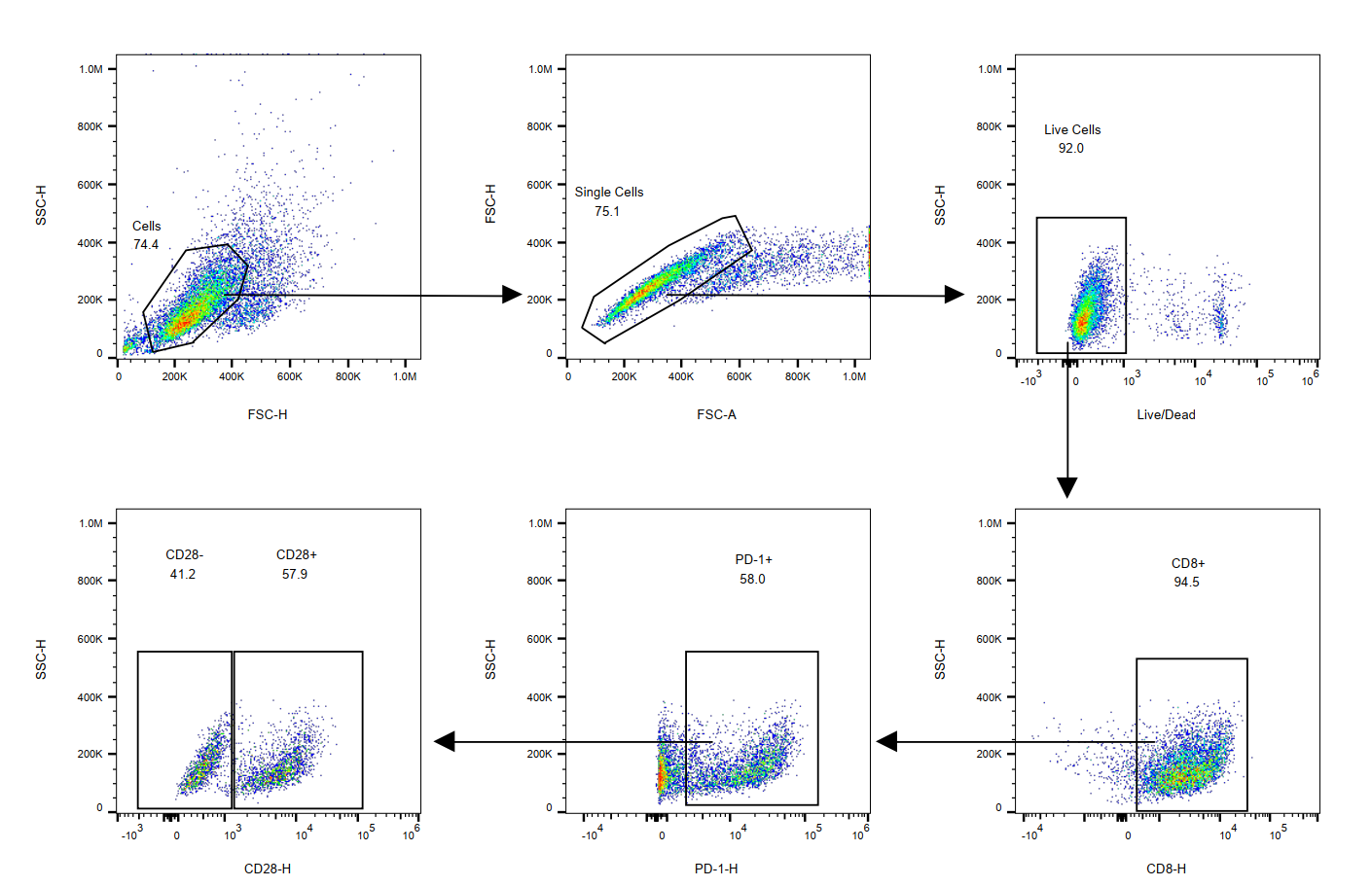


Gating strategies for Normal tissues


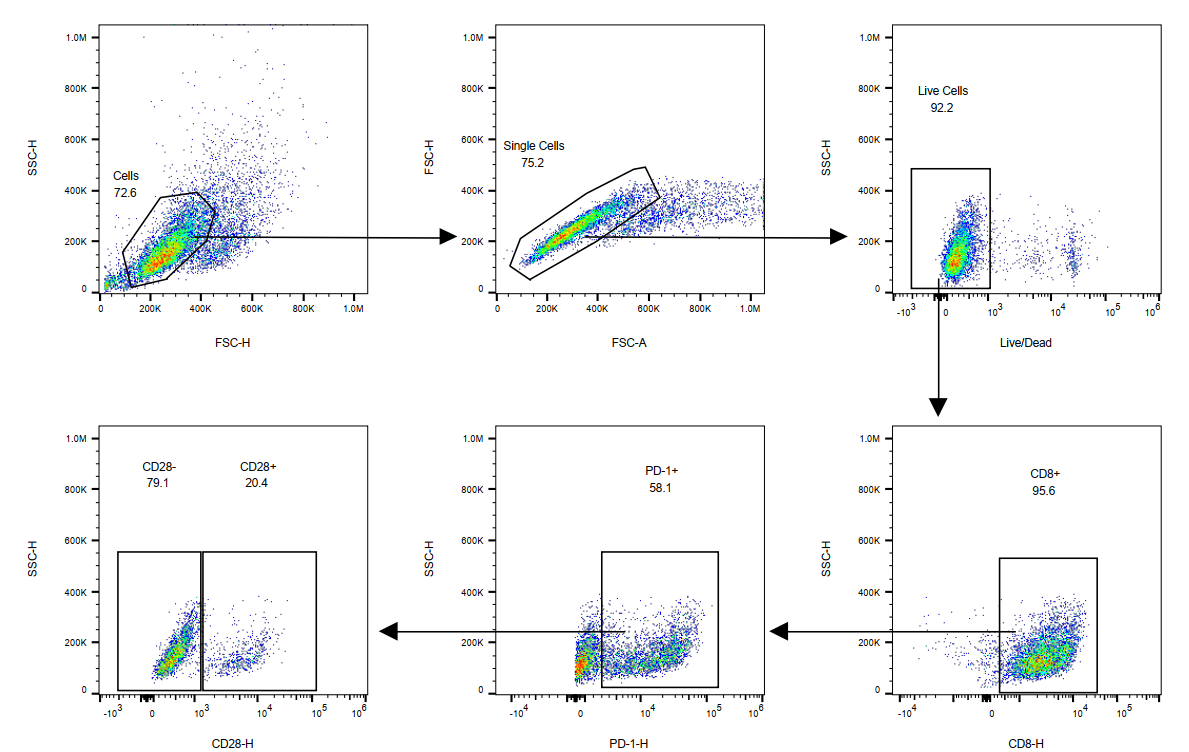


Gating strategies for HCC and Normal tissues
